# Malaria-driven adaptation of MHC class I in wild bonobo populations

**DOI:** 10.1038/s41467-023-36623-9

**Published:** 2023-02-23

**Authors:** Emily E. Wroblewski, Lisbeth A. Guethlein, Aaron G. Anderson, Weimin Liu, Yingying Li, Sara E. Heisel, Andrew Jesse Connell, Jean-Bosco N. Ndjango, Paco Bertolani, John A. Hart, Terese B. Hart, Crickette M. Sanz, David B. Morgan, Martine Peeters, Paul M. Sharp, Beatrice H. Hahn, Peter Parham

**Affiliations:** 1 Department of Anthropology, Washington University in St. Louis, Saint Louis, 63130 MO USA; 2 Department of Structural Biology, Stanford University School of Medicine, Stanford, CA 94305 USA; 3 Department of Medicine, Perelman School of Medicine, University of Pennsylvania, Philadelphia, PA 19104 USA; 4 Department of Microbiology, Perelman School of Medicine, University of Pennsylvania, Philadelphia, PA 19104 USA; 5 Department of Ecology and Management of Plant and Animal Resources, Faculty of Sciences, University of Kisangani, BP 2012 Kisangani, Democratic Republic of the Congo; 6 Institute of Human Sciences, School of Anthropology and Museum Ethnography, University of Oxford, Oxford, UK; 7 Frankfurt Zoological Society, Lomami National Park Project, Kinshasa, Democratic Republic of the Congo; 8 Congo Program, Wildlife Conservation Society, Brazzaville, Republic of the Congo; 9 Lester E. Fisher Center for the Study and Conservation of Apes, Lincoln Park Zoo, Chicago, IL 60614 USA; 10 Recherche Translationnelle Appliquée au VIH et aux Maladies Infectieuses, Institut de Recherche pour le Développement, University of Montpellier, INSERM, 34090 Montpellier, France; 11 Institute of Ecology and Evolution, University of Edinburgh, Edinburgh, EH9 3FL UK; 12 Centre for Immunity, Infection, and Evolution, University of Edinburgh, Edinburgh, EH9 3FL UK; 13 Department of Microbiology & Immunology, Stanford University School of Medicine, Stanford, CA 94305 USA

**Keywords:** Genetic variation, Ecological genetics, Population genetics

## Abstract

The malaria parasite *Plasmodium falciparum* causes substantial human mortality, primarily in equatorial Africa. Enriched in affected African populations, the B*53 variant of HLA-B, a cell surface protein that presents peptide antigens to cytotoxic lymphocytes, confers protection against severe malaria. Gorilla, chimpanzee, and bonobo are humans’ closest living relatives. These African apes have *HLA-B* orthologs and are infected by parasites in the same subgenus (*Laverania*) as *P. falciparum*, but the consequences of these infections are unclear. *Laverania* parasites infect bonobos (*Pan paniscus*) at only one (TL2) of many sites sampled across their range. TL2 spans the Lomami River and has genetically divergent subpopulations of bonobos on each side. Papa-B, the bonobo ortholog of HLA-B, includes variants having a B*53-like (B07) peptide-binding supertype profile. Here we show that B07 Papa-B occur at high frequency in TL2 bonobos and that malaria appears to have independently selected for different B07 alleles in the two subpopulations.

## Abstract (web summary)

A variant of MHC class I is protective against severe malaria disease and enriched in affected African populations. Here, Wroblewski et al., characterise the consequences of malaria infection in wild bonobo populations showing that the presence of malaria drives a similar evolution in immune genes.

## Introduction

At least six species of *Plasmodium* parasites commonly infect humans, causing substantial morbidity and mortality worldwide. In 2021, ~247 million infections led to ~619,000 deaths from malaria^1^. About 96% of deaths from malaria occur in Africa and are due to *Plasmodium falciparum*^1^. Over recent human history malaria, especially that caused by *P. falciparum*, has exerted a considerable selection pressure on the human genome^2,3^. While many of the effects reported concern genetic variants affecting the structure or function of red blood cells^2,3^, loci from the Major Histocompatibility Complex (MHC) have also been implicated^4^. *HLA-B*, the most polymorphic human MHC class I gene^5^, encodes membrane glycoproteins that bind peptide antigens derived from the proteins of intracellular pathogens^6^. These peptide:MHC-B complexes activate natural killer (NK) cells of innate immunity and cytotoxic T-cells of adaptive immunity^7,8^. Various associations have been suggested between HLA variants (allotypes) and the severity of malaria in humans (reviewed by Sanchez-Mazas^4^). In particular, the HLA-B*53 allotype was reported to associate with protection from the worst effects of *P. falciparum* infection in a Gambian population in West Africa^9^. Strikingly, HLA-B*53 frequencies across African populations are strongly positively correlated with the local prevalence of *P. falciparum* malaria, suggesting that this allotype has undergone pathogen-driven selection^10^. A candidate protective mechanism is that HLA-B*53 presents peptides cleaved from *P. falciparum* liver stage proteins, which activate cytotoxic CD8 + T cells^11^. A protective effect of HLA-B*53 could also involve an NK cell response to *P. falciparum* mediated by the interaction of its Bw4 epitope with the KIR3DL1 NK cell receptor^12^.

**Fig. 1:**
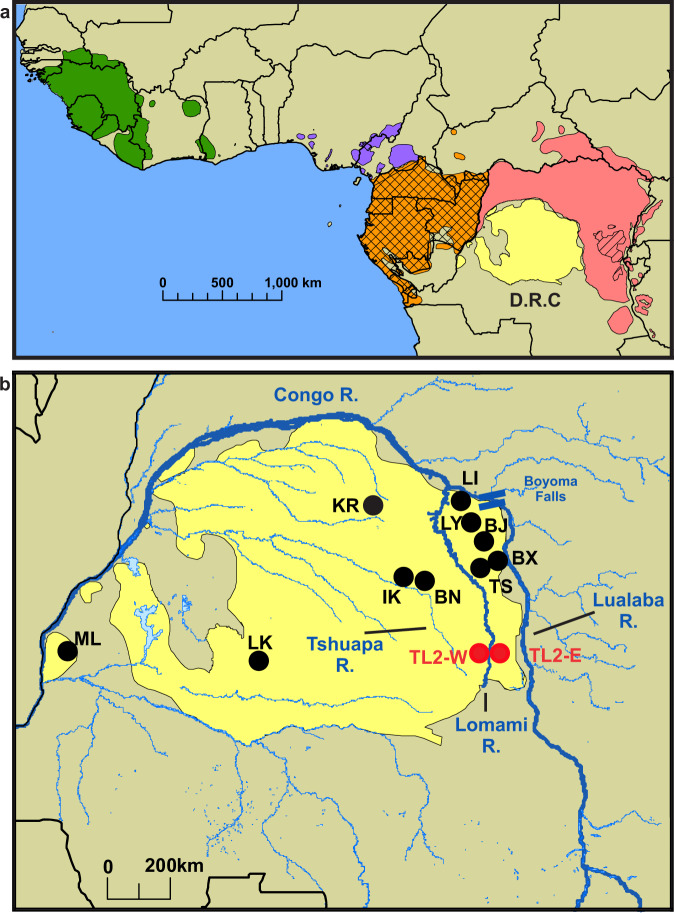
African ape ranges, bonobo study sites, and presence of *Laverania*. **a** The ranges of bonobos (*Pan paniscus*, yellow), western chimpanzees (*Pan troglodytes verus*, green), Nigeria-Cameroonian chimpanzees (*P. t. ellioti*, purple), central chimpanzees (*P. t. troglodytes*, orange), eastern chimpanzees (*P. t. schweinfurthii*, red), western gorillas (*Gorilla gorilla*, cross hatch), and eastern gorillas (*Gorilla beringei*, single hatch) are shown. *Laverania* parasites have been detected in all African ape species, except the Eastern gorilla. Range data were obtained from the IUCN Red List^70–73^. **b** The bonobo range (yellow) is shown in relation to the Congo, Lualaba, and Lomami Rivers (thick dark blue lines). Parallel lines mark the Boyoma Falls, a potential crossing point of the bonobo common ancestor^24,25^. Two-letter codes indicate the bonobo study sites. TL2-W bonobos are physically separated from TL2-E bonobos by the Lomami River. TL2 bonobos on both sides of the river are endemically infected with *Laverania* (red circles)^14^. One faecal sample collected at KR contained *P. lomamiensis*^14^.

The African great apes—chimpanzees, bonobos, and gorillas—are the closest living relatives of humans (Fig. 1a). Molecular epidemiological surveys of wild African apes have identified infections by at least 12 distinct *Plasmodium* species, seven of which fall within the same subgenus, *Laverania*, as *P. falciparum*^13–17^. Among wild chimpanzees and gorillas, young and pregnant apes appear more susceptible to infection^18–21^. Very few cases of apparent malarial disease have been reported in apes^22^, and the pathogenicity of *Plasmodium* in wild apes remains unclear. No host mutations conferring resistance to infection have been identified^23^. The pattern of *Laverania* infection differs among the ape species. In chimpanzees (*Pan troglodytes*) and western gorillas (*Gorilla gorilla*) infections are found across much of their ranges, whereas *Laverania* have not been detected in any samples from wild eastern gorillas (*Gorilla beringei*)^13^. In contrast, bonobos (*Pan paniscus*) were found to be commonly infected with *Laverania* at a single location, the Tshuapa-Lomami-Lualaba (TL2) study site, where 38% of bonobos had detectable parasite DNA in their faeces, whereas only one positive sample was found among more than 1400 analyzed from 10 other sites across their natural range^14^. This absence of *Plasmodium* from most bonobo field sites could not be explained by parasite seasonality or differences in the abundance of plant or microbiome constituents identified in faeces^14^. At TL2, separate bonobo populations live on the east (TL2-E) and west (TL2-W) banks of the Lomami River (Fig. 1b). Bonobos are thought to have originated in the northeastern part of their present-day range, having crossed the Congo River from the north, then splitting into eastern and western subpopulations on either side of the Lomami River^24,25^. The river continues to inhibit gene flow between bonobos at TL2-E and TL2-W, as seen from analyses of mitochondrial DNA haplotypes^14,26^ and craniodental morphology^27^, but does not prevent transmission of *Plasmodium* since bonobos on both sides of the river at TL2 are infected by similar strains of parasites^14^.

Bonobos at TL2 are predominantly infected by two species of *Laverania*^14^. One, *P. lomaniensis*, only infects bonobos. The close relationship of *P. lomaniensis* to *P. reichenowi* (from chimpanzees) and *P. praefalciparum* (from gorillas) mirrors the phylogenetic relationships of the three host species and suggests that these parasites may have co-evolved with their hosts^14^. The second species, *P. gaboni*, is a common infection of chimpanzees, and the parasite strains in bonobos appear to have been derived from the eastern subspecies of chimpanzee (*P. t. schweinfurthii*) after their split from the central subspecies (*P. t. troglodytes*)^14^. Thus, ancestral bonobos may have been infected by *P. lomamiensis*, which was maintained in bonobos east of the Lomami, but lost from populations to the west of the river. In contrast, *P. gaboni* appears to have been acquired by eastern bonobos more recently^14^. Both parasite species likely spread from TL2-E to bonobos at TL2-W.

The uniquely contrasting phylogeographies of bonobos and *P. lomamiensis* enable a test of malaria-driven natural selection in genetically divergent bonobo populations living on opposite sides of the Lomami River. African apes have orthologues of the *HLA-B* locus^5^. From the known peptide-binding specificities of HLA-B allotypes, de Groot et al. predicted^28^ the specificities for bonobo MHC-B (Papa-B) allotypes identified in captive and wild bonobos. These investigators grouped the *Papa-B* alleles into supertypes^29^ comprised of alleles with similar specificity^28^. Among the 32 *Papa-B* alleles characterized, 16 grouped with the human B07 supertype, which includes HLA-B*53, 13 grouped with the human B27 supertype, and the remaining three were similar to a chimpanzee MHC-B supertype (Patr-B*17:03) that has no human counterpart^28^. The finding that 50% of Papa-B allotypes are of the B07 supertype was unexpected and led de Groot et al. to hypothesize that selection from *Laverania* had increased the frequency of Papa-B allotypes with an HLA-B*53-like peptide binding specificity^28^. However, in contrast to the positive correlation between the frequency of HLA-B*53 and the prevalence of *P. falciparum* observed among sub-Saharan Africans^10^, de Groot et al. suggested^28^ that the high frequency of the B07 supertype in bonobos could explain the absence of *Plasmodium* seen in the majority of bonobo populations.

Here we examined MHC-B polymorphism in seven well-sampled populations of wild bonobos, including the first characterisation of *Papa-B* alleles at TL2. We show that B07 supertype MHC-B alleles have a higher frequency in both bonobo populations at TL2 compared to other sites. This observation mirrors the selection for HLA-B*53 among *P. falciparum*-infected populations of sub-Saharan Africans^10^ and indicates that the presence of B07 MHC-B cannot explain the lack of *Laverania* parasites across most of the bonobo range west of the Lomami. Instead, the preponderance of B07 allotypes at TL2 suggests that they are providing protection against severe disease, as was suggested for HLA-B*53 among Gambians^9^. Remarkably, the specific B07 alleles found at high frequency differ between TL2-E and TL2-W, reflecting the prior genetic divergence between these two bonobo populations^14,26^. This is consistent with the presence of *Laverania* independently selecting for the B07 supertype at the two sites and implies that infection with these parasites is detrimental to bonobo fitness.

## Results

### *Papa-B* allele frequencies differ among bonobo populations

We previously determined the sequences of *Papa-B* alleles from 108 bonobos from five sites (ML, LK, IK, BN, KR)^30^, all located west of the Lomami River (Fig. 1b), and (almost) all completely lacking evidence of *Plasmodium* infection (one of 69 samples at KR was positive)^14^. In this study, we characterized *Papa-B* alleles using 116 samples collected from 59 bonobos from two divergent populations present on the two sides of the Lomami River at TL2 where *Plasmodium*, and especially *Laverania*, infections are common^14^. TL2 is an established research site where extensive sample collection was possible during field surveys. In addition, we examined available samples from five other locations east of the Lomami, which do not represent established field sites (Fig. 1b). These samples, which were fewer in number and collected opportunistically, yielded data for another seven bonobos (discussed below). Sequence variation among *Papa-B* alleles is concentrated in exons 2 and 3 which encode the peptide-binding site, where this variation modulates Papa-B interactions with peptides, T-cell receptors, and the Killer cell Immunoglobulin-like Receptors (KIR) of Natural Killer (NK) cells^31,32^. Separate PCR reactions were used to amplify and sequence exons 2 and 3 of *Papa-B*^30,33^. The number of individual bonobos was determined from their composite genotypes, including *Papa-B* as well as microsatellite and mitochondrial D-loop sequences that were characterized previously^14,15,30,34^. These data indicated a minimum of 52 TL2-E and seven TL2-W bonobos are represented in this study (Table 1).

**Table 1:** Number *Papa-B* homozygotes and heterozygotes per study site

	West of Lomami						East of Lomami					
Number of	ML	LK	IK	BN	KR	TL2-W	TL2-E	TS	BX	BJ	LY	LI
Faecal samples	46	37	36	67	67	15	101	2	1	2	17	1
Bonobos (Min)	14	17	18	22	37	7	52	2	1	2	1	1
*Papa-B* alleles	3	10	11	13	14	9	9	3	1	2	2	1
Heterozygotes	6	15	16	20	29	7	43	1	0	2	1	0
Homozygotes	8	2	2	2	8	0	5^a^	U^b^	1	0	0	1

^a^The zygosity of four bonobos in TL2-E could not be determined.

^b^Bonobo with an unknown second allele.

**Fig. 2:**
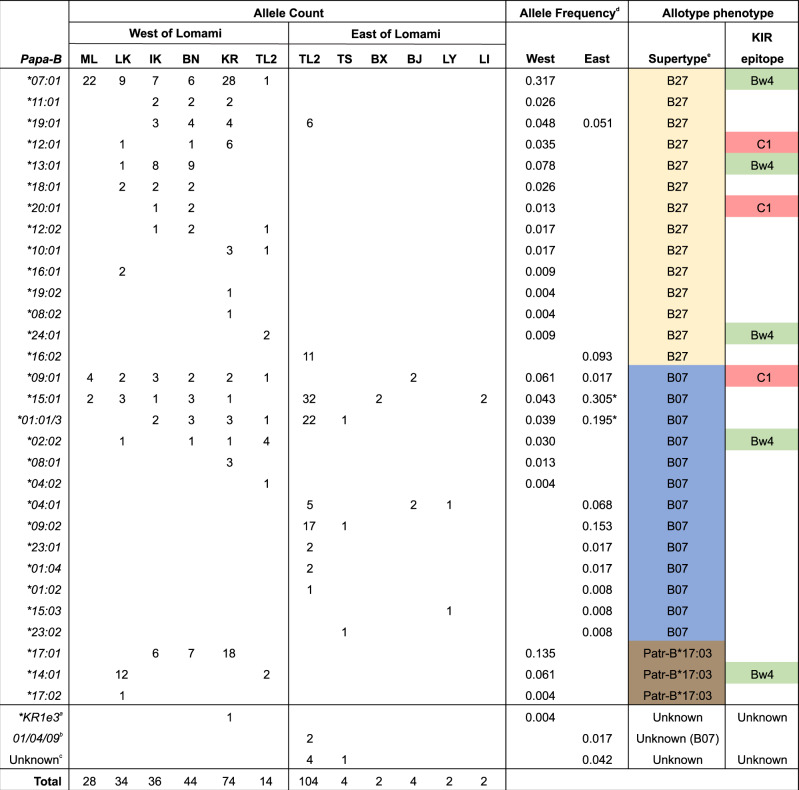
*Papa-B* allele counts, frequencies, and allotype phenotypes at 12 study sites. For each *Papa-B* allele, it is noted which peptide-binding supertype that it possesses (B27 (yellow), B07 (blue), Patr-B*17:03 (17:03, brown), and whether or not it encodes a KIR epitope (either Bw4 (green) or C1 (red)). ^a^KIR epitope and supertype are “Unknown.” ^b^Genotyping resolved the second allele as being one of three alleles (Papa-B*01:02, 04:01, 09:02), all of which have the B07 supertype and lack a KIR epitope. ^c^For five eastern bonobos, a second *Papa-B* allele could not be identified. ^d^Asterisks indicate significant frequency differences between bonobo populations west and east of the Lomami River (two-tailed Fisher’s exact tests, *p* < 0.0001; all comparisons in Supplementary Table 5). ^e^B07 phenotype distributions for each population are given in Supplementary Table 2.

Each of the TL2-W and TL2-E populations were found to have nine *Papa-B* alleles, each encoding a distinct Papa-B allotype; at the five previously characterized sample sites there were 3–14 alleles (Fig. 2, Supplementary Table 1). There was no evidence of deviation from expected Hardy-Weinberg genotype frequencies at either TL2-W (*p* = 0.65) or TL2-E (*p* = 0.31). Only two alleles found at TL2-E were also present in bonobos from west of the Lomami: Papa-B*01:01/3 was shared between TL2-W and TL2-E, while Papa-B*15:01 was not found at TL2-W but was present in other western populations (Fig. 2). In contrast, seven of the nine alleles at TL2-W were also found in other western populations (Fig. 2). Thus, TL2-W and TL2-E bonobos have largely non-overlapping subsets of *Papa-B* alleles, with TL2-W bonobos exhibiting much greater similarity to the other populations west of the Lomami than to TL2-E bonobos.

**Fig. 3:**
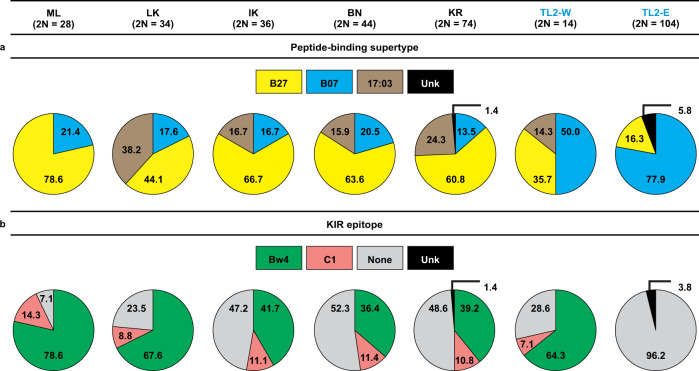
Frequencies of Papa-B alleles in bonobo populations. For each field site, the number of Papa-B allotypes is shown. Unknown (Unk) second Papa-B allotypes in TL2-E and KR are indicated in black (Fig. 2). Sites with endemic *Laverania* infection are shown in blue font. Source data are provided as a Source Data file. **a** Frequencies of the three Papa-B peptide-binding supertypes are shown: B27 (yellow), B07 (blue), Patr-B*17:03 (17:03, brown). B07 is more frequent in TL2-E and TL2-W than in any western population (two-tailed Fisher’s exact tests with Bonferroni adjusted alpha levels of 0.0167 per test: TL2-E, TL2-W, *p* = 0.011; TL2-E, Other-West *p* < 0.0001; TL2-W, Other-West, *p* = 0.007). **b** Population frequencies of KIR epitopes among Papa-B (Fig. 2). KIR epitopes (either Bw4 (green) or C1 (red)) are more frequent in all western populations compared to TL2-E, which has no epitopes (None, grey). (two-tailed Fisher’s exact tests with Bonferroni adjusted alpha levels of 0.0083 per test: all pairwise comparisons, *p* < 0.0001).

Among the five previously characterized western populations of bonobos, in the absence of *Plasmodium* infections, the most common *Papa-B* supertype was B27, at an average frequency of 62% (range 44% to 79%; Fig. 3a). In contrast, the predominant supertype in the presence of *Laverania* parasites at TL2 was B07, found at frequencies of 78% and 50% at TL2-E and TL2-W, respectively, compared to an average frequency of only 17% across the five previously characterized western populations (Fig. 2; Fig. 3a). Among the five western populations, the frequency of B07 did not vary significantly, but the differences in frequency of B07 in each pairwise comparison among TL2-E, TL2-W, and the combined five western populations are all statistically significant (two-tailed Fisher’s exact tests: TL2-E, TL2-W, *p* = 0.011; TL2-E, Other-West *p* < 0.0001; TL2-W, Other-West, *p* = 0.007) (Supplementary Table 3). Thus, compared to western bonobos, both TL2-E and TL2-W have substantially elevated frequencies of B07 supertype alleles. Remarkably, however, the particular B07 alleles differ between TL2-E and TL2-W (Fig. 2).

### Eastern bonobos at multiple sites encode the B07 supertype at high frequency

Previous work suggested that, early in their diversification, some bonobos crossed the Lomami, and that the river remains a barrier between the two subpopulations that arose from this separation^14,26,27^. The differences in *Papa-B* allele frequencies described above are consistent with this. However, the very high frequency of the B07 supertype at TL2-E derived from only a single well-sampled bonobo population from east of the Lomami. To address this, we analysed available samples from five additional sites east of the Lomami (Fig. 1). However, these sites (TS, BX, BJ, LY, and LI) are not established field sites where bonobos can be studied over time. Instead, samples were collected opportunistically during forest transects and confirmed to be of bonobo origin by mitochondrial DNA analysis. We previously characterized samples from two bonobos from BJ, and one from BX^30^. To increase the number of individuals from this part of the bonobo range, we examined 20 additional faecal samples from three sites northeast of TL2 (TS, LY, and LI) (Table 1). By generating microsatellite genotypes at 13 loci and determining the mitochondrial haplotype by sequencing the hypervariable D-loop^14,15,30,34^ (Supplementary Table 4), we found that they represent at least four additional bonobos (Table 1). We also screened the samples for *Laverania* infection, but no *Plasmodium* sequences were detected (Supplementary Table 4); however, given the small number of samples, this should not be taken as evidence of the absence of these parasites from these locations.

Among the seven bonobos from these five additional sites east of the Lomami there were seven *Papa-B* alleles, all belonging to the B07 supertype (Fig. 2; Supplementary Table 4). While the numbers are small, they are consistent with there being a high frequency of this supertype across eastern bonobo populations, and not just at the TL2 site. With these additional data, *Papa-B* has now been characterized for 174 wild-living bonobos sampled from 12 field sites, six on each side of the Lomami River (Fig. 1). A total of 31 alleles have been observed, of which only four are common to bonobos living on both sides of the river (Fig. 2). Two of the common alleles, Papa-B*15:01 and Papa-B*01:01/3, which both encode the B07 supertype, occur at significantly higher frequencies at TL2-E than in western populations (Fig. 2; Supplementary Table 5). This segregation of *Papa-B* alleles parallels that observed for their mitochondrial haplotypes, none of which are common to the eastern and western populations^14^ (Supplementary Table 6, Supplementary Fig. 1), which is consistent with an absence of gene flow across the Lomami^14,26^.

### A potential B07 target epitope is conserved among *Laverania*

**Fig. 4:**
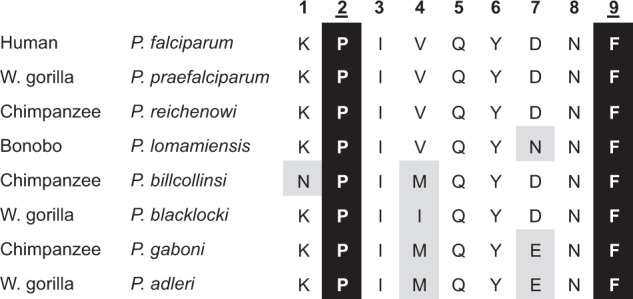
Limited variation in the LSA-1 ls6 epitope of *Laverania*. Shown are amino acid sequences of the nonamer ls6 peptide for LSA-1 proteins of eight *Laverania* species. Sequences are listed, along with their natural host, according to their phylogenetic relatedness^13,14^. Positions 2 and 9 (underlined) are the conserved anchor positions (highlighted black) that hold the peptide within the MHC-B peptide-binding groove. Grey highlights residues that differ from that of *P. falciparum*.

The B07 supertype is distinguished by its preference for a proline at position 2 (P2-P) of nonamer peptides^29^ (Supplementary Fig. 4). HLA-B*53, a B07 allotype, binds ls6, a P2-P-containing nonamer derived from the *P. falciparum* liver-stage antigen-1 (LSA-1), and presents this peptide to cytotoxic T-cells^11^. HLA-B*53-mediated protection from severe malaria^9^ could arise from T-cells responding to expression of the liver stage antigen and reducing parasite spread to erythrocytes. The ls6 peptide is conserved among strains of *P. falciparum*^35,36^. Orthologues of the gene encoding LSA-1 are found in the genomes of the *Laverania* parasites of African apes. Using single genome amplification^14–16^, we generated partial *LSA-1* sequences encompassing the region encoding the ls6 peptide for three samples of *P. lomamiensis* (the bonobo-specific parasite), as well as 18 other *Laverania* from chimpanzees and gorillas, and compared the sequences with those available from five complete genomes^37^ (Supplementary Table 7). We found no intraspecific polymorphism in the nonamer sequence. Among the two species that commonly infect bonobos, *P. lomamiensis* and *P. gaboni*, the ls6 epitopes differ from that of *P. falciparum* at one and two residues, respectively, but the P2-P and P9-F anchor residues within the nonamer are conserved across all *Laverania* (Fig. 4). Thus, this conserved peptide could be a target for anti-parasite responses mediated by *Papa-B* alleles of the B07 supertype.

### Absence of KIR epitopes in TL2-E bonobos

Whereas all MHC-B allotypes engage CD8^+^ T-cell receptors, only subsets have either a Bw4 or a C1 epitope that are recognized by different lineages of KIR^32^. In humans, HLA-B*53 has a Bw4 epitope, and it has been suggested that the protection against *P. falciparum* could involve an NK cell response mediated by an interaction between Bw4 and KIR3DL1^12^. Among *Papa-B* alleles, the presence of KIR epitopes is not associated with a particular supertype (B07 vs non-B07, odds ratio = 1.18, 95% CI = 0.32–4.42, *p* = 0.80) (Supplementary Table 8). Across all study populations, only two B07 Papa-B allotypes have a KIR epitope: Papa-B*02:02 has Bw4 and Papa-B 09:01 has C1 (Fig. 2). Both of these allotypes are found at TL2-W, where Papa-B*02:02 is the most frequent allotype, but neither has been detected at TL2-E (Fig. 2). In fact, none of the nine different allotypes found at TL2-E contained a KIR epitope, whereas all six well-sampled western populations have both Bw4 and C1 allotypes (Fig. 3b). This difference in the frequency of KIR epitopes between TL2-E and the western populations is significant (two-tailed Fisher’s exact tests: TL2-E, all pairwise comparisons, *p* < 0.0001) (Supplementary Table 3). However, KIR epitopes carried by Papa-B are not entirely absent from eastern populations because two of the four alleles at BJ were Papa-B*0901 which encodes a B07 allotype with the C1 epitope (Fig. 2).

## Discussion

The consequences of *Laverania* parasite infection for wild ape populations have been difficult to assess, given the biology and ecology of both parasites and apes, the latter of which can only be studied non-invasively^23^. From comparison of Papa-B polymorphism at field sites throughout the bonobo range, we obtained evidence suggesting that selection was imposed by malaria parasites. The TL2 site proved particularly informative because it has bonobo populations on each bank of the Lomami River. These populations evolved independently and are genetically^14,26^ and morphologically^27^ distinct. Despite their physical separation, both TL2 populations are *Laverania* infected because the river does not impede mosquito-driven transmission of the parasite^14^. The B07 peptide-binding supertype of Papa-B has high frequencies in TL2-E and TL2-W, but much lower frequencies in the five well-sampled western populations where *Laverania* infections are rare or absent^14^. It is generally accepted that MHC polymorphisms must be maintained by selection^38,39^. Moreover, over the timescale of the divergence of the eastern and western bonobo subpopulations^26^ the great majority of neutral polymorphisms in their ancestors are expected to have drifted to fixation or loss^40^, given the estimated effective population sizes of these apes^41^. Although the sampled populations are small, due to the requirement of using non-invasive approaches, our results are consistent with pathogenic *Laverania* infection having driven the evolution of *Papa-B* in TL2 bonobos.

Despite the initial results in The Gambia^9^, subsequent studies of HLA polymorphism in other malaria-endemic human populations did not replicate evidence of the protective association of HLA-B*53^42–44^. Genome-wide association studies (GWAS) also failed to identify *HLA* genes as under selection by malaria^45–48^. However, the extreme polymorphism of classical, antigen-presenting class I *HLA* genes presents challenges for GWAS because *HLA* genes are generally underrepresented on GWAS chips, and GWAS would not be expected to capture HLA protein phenotypes under selection that span multiple alleles and/or that arise from combinations of changes at several amino acids^49^. The most compelling support for an advantage conferred by HLA-B*53 is the observation that its frequency across 40 African populations is strongly positively correlated with the prevalence of *P. falciparum*^10^. Thus, while there may be population-specific variation in the association of HLA-B*53 with protection against severe malaria, this correlation suggests that HLA-B*53 has undergone pathogen-driven selection in sub-Saharan Africa. In the same study^10^, HLA-B*78 was also positively correlated with the prevalence of *P. falciparum*, albeit less strongly than HLA-B*53. Both HLA-B*53 and HLA-B*78 have the B07 supertype, suggesting that there is an advantage conferred by allotypes with this binding specificity. The increased frequency of B07-supertype Papa-B allotypes in bonobo populations endemically infected with *Laverania* parallels the correlation between the frequency of the HLA*B53 and HLA-B*78 B07 allotypes in African populations that are endemically infected with *P. falciparum*^10^. This points to *Papa-B* alleles of the B07-supertype being selectively present in affected bonobo populations because they provide protection against malaria-like disease in a manner comparable to the two B07 HLA-B allotypes^9^. There are likely many peptide antigens with a P2-P anchor that would be targeted by a B07 immune response. For humans, one *P. falciparum* target is the LSA-1 protein^11^, which is expressed only at the liver stage^36^. The ls6 epitope of LSA-1 has the P2-P anchor of peptides presented by B07-supertype HLA-B and elicits strong T-cell responses in humans having HLA-B*53^11^. Such a response to a liver stage antigen could reduce the parasite burden spreading to erythrocytes. Because the ls6 epitope and its P2-P anchor are conserved among all *Laverania* species, this epitope is a potential target for a response by CD8^+^ T-cells restricted by B07-supertype Papa-B.

It has been suggested that the protective effect of HLA-B*53 involves an NK cell response to *P. falciparum* that is mediated by the interaction of its Bw4 epitope with NK cells expressing KIR3DL1^12^. HLA class I is expressed on hepatocyte cell surfaces where it has the potential to interact with KIR expressed by liver-resident NK cells and modulate NK cell activity^50^. Binding between Bw4 on HLA-B expressed by a healthy cell and KIR3DL1, an inhibitory receptor, prevents the NK cell-mediated destruction that can occur when infection results in the downregulation of HLA-B^50^. However, it is unknown whether HLA-B, or other MHC class I, expression is downregulated in cells infected by *P. falciparum*. In humans, the frequencies of both HLA-B*53 or HLA-B*78 are positively correlated with *P. falciparum* prevalence, but the correlation was stronger for HLA-B*53^10^. While both allotypes have the B07 supertype, HLA-B*53 also has the Bw4 epitope that engages the NK cell receptor KIR3DL1 whereas HLA-B*78 does not. This pattern is consistent with the B07 peptide-presenting specificity resulting in the activation of CD8^+^ T-cells as the main defense against *P. falciparum* via HLA-B but suggests that an NK response mediated by the ligation of Bw4 and KIR3DL1 contributes additional protection. Among bonobos, both TL2-W and TL2-E have a high frequency of Papa-B with the B07 supertype, and TL2-W has some B07-supertype Papa-B allotypes that also have a KIR epitope. However, the latter is not the case for TL2-E Papa-B. Thus, in contrast to humans, the B07 peptide-presenting specificity appears to be the main component of the anti-*Laverania* defense mediated by Papa-B, with NK cell recruitment via Papa-B in response to malaria infection not required.

The timeframe over which *Laverania* parasites have infected bonobos is not known with certainty. However, we have noted that the phylogenetic relationships among *P. lomamiensis* and its two close relatives infecting chimpanzees and gorillas mirror that of the apes and is consistent with co-divergence of these parasites with their hosts^14^. Ancestral bonobos are thought to have originated in the northeastern part of their present-day range, having crossed the Congo River near the Boyoma Falls at a time when the river there was relatively shallow^24,25^ (Fig. 1b). Under the co-divergence scenario, this ancestral bonobo population would have been infected with a recent ancestor of *P. lomamiensis*, and the subpopulation of bonobos that remained east of the Lomami River, including the ancestors of the bonobos at TL2-E, would be predicted to have remained endemically infected to the present day. More recently, these eastern bonobo populations acquired a second *Laverania* species, *P. gaboni*, from nearby eastern chimpanzees via mosquitoes that crossed the Lualaba River^14^ (Fig. 1). This long-term exposure to *Laverania* is then a plausible explanation for the very high frequency (78%) of B07 supertype *Papa-B* alleles in bonobos at TL2-E. Although we sampled only seven bonobos at other locations east of the Lomami, all seven were homozygous for B07 alleles, suggesting that the TL2-E population is typical of others in the eastern region. From comparisons of mtDNA haplotypes it has been suggested that western bonobos arose about 0.26–0.95 million years ago, when their ancestors crossed the Lomami in the north of their range^26^. Since all western bonobo populations from widespread locations appear to lack *Laverania* infections^14^, it seems likely that malaria parasites were lost at, or soon after, the origin of the western populations. MHC polymorphisms are exceptionally long-lived, and it is often inferred that this results from some form of balancing selection, with alternative alleles favoured due to resistance to diverse pathogens^51^. In the absence of *Laverania*, the selection favouring the B07 alleles would be missing, and alternative alleles conferring resistance to other (as yet unidentified) pathogens would be selected to higher frequencies. Across the western populations shared alleles of the B27 supertype are present at high frequencies (44–79%) while B07 alleles occur at frequencies at or below 20%. Among bonobos west of the Lomami, the exception is the population at TL2-W. These bonobos have regained *Laverania* infections^14^, but presumably only after they migrated to this location within range of the mosquitoes that bite bonobos at TL2-E. At TL2-W the frequency of B07 alleles has climbed to about 50% and, strikingly, most of these B07 alleles are identical to those found at low frequencies in other western populations, but different from those found at high frequencies at TL2-E. That the two TL2 populations on either side of the river have clearly independently evolved high frequencies of B07 alleles points to the location as being important in providing the selective force, and the only relevant shared feature of TL2-W and TL2-E bonobos that we are aware of is the presence of *Laverania* infections. In turn, the observation that long-term exposure to *Laverania* during the ancestry of the TL2-E population has led to a frequency of B07 alleles as high as 78% suggests that malaria parasites have exerted stronger selective pressure than any other pathogens at that site.

The skewed distribution of B07-supertype Papa-B in wild bonobos implies that *Laverania* infection has had deleterious consequences for bonobos. In humans, the haemolysis of infected erythrocytes is the main mechanism that leads to the typical signs of fever, chills, headache, anaemia, and gastrointestinal symptoms^52^. However, it is stiffening of infected erythrocyte cell membranes that can lead to capillary obstruction and the development of severe malaria, which includes severe anaemia and multi-organ damage including that of the brain (cerebral malaria)^52^. Pregnant women are particularly vulnerable facing increased risk of death and other poor outcomes^52^. The observation that young and pregnant chimpanzees and gorillas appear more susceptible to infection^18–20^ raises the possibility of a similar pathology in African apes. It will thus be essential to investigate bonobos and other apes at long-term study sites where detailed, daily observational data and regular, rather than opportunistic, sample collection from known individuals will facilitate a better understanding of the natural history of *Laverania* infection and its fitness consequences. Since in humans HLA-B*53 and the B07 supertype have been associated with other infectious diseases^53–56^, it will also be important to conduct more in-depth surveys of additional pathogens that could be influencing selection of Papa-B in bonobos. However, simian immunodeficiency virus (SIV), which is significantly associated with *Patr-B* alleles in wild chimpanzees^33^, can be excluded since bonobos are not naturally infected with this virus^34,57^. Finally, it will be important to characterize *Laverania* prevalence and Papa-B polymorphisms in additional bonobo populations along the two banks of the Lomami, particularly in the purported bonobo ancestral region northeast of the river, to better define the range of infection and its selective effects among bonobo populations.

## Methods

### Bonobo samples

All genetic work was performed using banked faecal samples that were collected non-invasively (Table 1). Therefore, this work is not classified as animal research by the Stanford Administrative Panel on Laboratory Animal Care or the Institutional Animal Care and Use Committees at Washington University in St. Louis and the University of Pennsylvania. At the time of collection, all samples were obtained with permission from the Ministries of Scientific Research and Technology, the Ministries of Health and Environment, and the National Ethics Committee, and the Department of Ecology and Management of Plant and Animal Resources at the University of Kisangani as previously reported^14,34^. We studied wild-living bonobos at 12 field sites, with six located west (Malebo, ML; LuiKotale, LK; Ikela, IK; Balanga, BN; Kokolopori, KR; Tshuapa-Lomami-Lualaba-West, TL2-W) and six located east (Lokoli forest, LI; Lobaye forest, LY; Bayandjo, BJ; Bananjale, BX; Tshuapa-Lomami, TS; Tshuapa-Lomami-Lualaba-East, TL2-E) of the Lomami River, respectively (Fig. 1b). Sex of the sample donors was not known because faecal samples were opportunistically collected during forest transects, however there is no expectation of a sex bias in the *Papa-B* population genetic data. Faeces were collected into RNAlater (1:1 vol/vol) for preservation. Samples were first stored and transported at ambient temperature and subsequently stored at −80 °C after reaching the U.S.A. Samples were shipped in compliance with the regulations of the Convention on International Trade in Endangered Species of Wild Fauna and Flora and with governmental export and import permits from the D.R.C. and U.S.A. Faecal DNA was extracted using the QIAamp Fast DNA Stool Mini Kit (Qiagen) and the QIAcube (Qiagen) robotic workstation according to the manufacturer’s instructions.

### Individual identification

The minimum number of bonobos at three additional eastern sites (TS, LY and LI) was determined by amplifying mitochondrial (D loop) DNA and microsatellite loci from faecal samples (Table 1, Supplementary Table 4) and by determining their genotype as reported previously for the other nine field sites (ML, LK, IK, BN, KR, TL2-W, TL2-E, BX, and BJ)^14,15,30,34^. Faecal samples were genotyped at 13 polymorphic, autosomal microsatellite loci (Supplementary Table 4) as described^58^ (see Supplementary Methods). Also genotyped to aid in individual identification of these sample donors was the amelogenin gene, which has an X and Y chromosome-specific allele that can be used for sex determination (males are heterozygotes and females are homozygotes)^59^. Mitochondrial D-loop sequences were amplified and sequenced as described^58^ with some modifications (see Supplementary Methods). Sequences were analysed using Geneious Prime 2022.0.2 (https://www.geneious.com). Three new mitochondrial haplotypes were obtained (Supplementary Fig. 1) (GenBank accession numbers: ON936815-ON936817).

### Papa-B *exon 2 and exon 3 genotyping*

110 bonobos at six field sites (ML, LK, IK, BN, KR, BJ) were genotyped previously for *Papa-B*^30^ (Table 1), while 64 bonobos (137 faecal samples) at six additional locations (TL2-W, TL2-E, TS, BX, LY, LI) were genotyped in the current study (see Supplementary Methods) (Table 1). The number of individuals determined by microsatellite analysis was confirmed, except for two instances where *Papa-B* genotyping differentiated closely related individuals with otherwise identical microsatellite genotypes (Supplementary Table 1). For samples from TL2-W, TL2-E, and BX, exons 2 and 3 of the *Papa-B* gene, which encode the polymorphic α1 and α2 domains of the peptide-binding site, were amplified and sequenced as described^30,33^. The sequences obtained were compared to all *Papa-B* alleles in the IPD-MHC database^60^ (https://www.ebi.ac.uk/ipd/mhc/group/NHP/) to identify the respective alleles.

Samples from TS, LY, and LI were genotyped using an NGS-adapted PCR (see Supplementary Methods). *MHC-B* exon 2 and exon 3 were amplified using locus-specific primers (Supplementary Table 10), but with MiSeq-specific adapters added to their 5’ end. PCR amplifications were performed in triplicate and MiSeq sequenced. FASTQ read pairs were sorted by index, and read pairs were assembled by PEAR 0.9.11^61^ (https://cme.h-its.org/exelixis/web/software/pear/). A de novo assembly of the read pairs was done in Geneious Prime 2022.1.1 (https://www.geneious.com) to obtain contigs. Contigs were then compared against known *Papa-B* alleles to determine whether there was an exact or close match. Analysis of the sample from BX was repeated using this newer method because it had a homozygous genotype (Supplementary Table 1), but this failed to yield additional *Papa-B* alleles. For other samples with suspected allelic dropout, PCRs were repeated, and amplicons were directly sequenced to confirm the genotype (Supplementary Table 4). The single homozygous sample (LI5125) was also subjected to the Patr-B*17-specific PCR as described above, but no B*17 lineage alleles were amplified.

### Papa-B *allele calling*

For each sample, exon 2 and exon 3 genotypes were compared for phase and identification of alleles, as described^30,33^. When previously characterized alleles were obtained for both exon 2 and 3, they were assumed to represent the known allele. For heterozygous individuals with a potential new allele, the exon 2 and 3 pair representing a previous allele was assumed to represent a known allele, with the second allele being defined by the other exons. Three of six new alleles were further verified by their detection in samples from other individuals. The other three alleles were identified in heterozygotes where the other exon pairing was able to be assigned to a known allele. Sequences of the six new alleles are deposited in GenBank (MW039484-MW039487, ON936818-ON936819) and named by the curators of the IPD-MHC database^60^ (https://www.ebi.ac.uk/ipd/mhc/group/NHP/). Since Papa-B*01:01 and *01:03 could not be distinguished by their exon 2 and 3 sequences, we used *01:01/3* for its identifier (Fig. 2, Supplementary Table 1, Supplementary Table 4). One exon 3 sequence previously reported as Papa-B*21:01 (GenBank KX786195)^30^ was different from Papa-B*21:01 in the IPD-MHC database^60^, and we are thus referring to the former as Papa-B*KR1e3.

For five bonobos (TL2-E: N = 4, TS: N = 1), the second *Patr-B* allele in the genotype was designated “Unknown” (Supplementary Table 1, Supplementary Table 4) since only single samples were available and ambiguous sequences were observed in at least two PCR reactions per exon. Two other bonobos, in TL2-E (TL2-07 and TL2-40.1), were heterozygous for exon 2 of Papa*B-01:01/3 and an exon 2 sequence shared by Papa-B*01:02, 04:01, 09:02, all three of which were otherwise present among TL2-E bonobos. Both bonobos were homozygous for Papa-B*01:01/3 in exon 3, but we could not resolve whether this was due to a PCR failure or the presence of a recombinant allele. We have thus designated this allele as “Papa-B*01:02/04:01/09:02”.

### Plasmodium screening of faecal samples

Bonobo faecal samples from the TS, LY, and LI field sites were screened for *Laverania* parasites as described^14,16^ (see Supplementary Methods).

### MHC-B supertype assignment

The repertoire of peptide antigens bound by an MHC-B allotype is determined by polymorphism at numerous variable positions in the α_1_ and α_2_ domains that form the peptide binding site. The site comprises six pockets (A-F), of which the B and F pockets bind anchor residues at positions 2 and 9 of a nonamer peptide antigen (Supplementary Fig. 4). Similarity in peptide-binding specificity is used to group subsets of HLA-B allotypes into peptide-binding supertypes^28,29^. Because no peptide-binding specificities for Papa-B have been determined experimentally, we used MHCcluster (v. 2.0)^62^ to predict peptide binding motifs for 33 Papa-B sequences deposited in the IPD-MHC database^60^ (https://www.ebi.ac.uk/ipd/mhc/group/NHP/) as of July 2022, including six Papa-B allotypes newly identified in this study. We also included 85 chimpanzee Patr-B from IPD-MHC and HLA-B allotypes representing the six human supertypes (B58, B07, B27, B44, B08, B62)^29^. Assignment of MHC-B allotypes to supertypes was based on clustering with HLA-B of known supertype (Supplementary Fig. 2, Supplementary Fig. 3), the sequence similarity within the B and F pockets, and the predicted amino acid preferences at P2 and P9 (Supplementary Fig. 4). Our assignments identified three supertypes: Papa-B similar to human HLA-B B07, Papa-B similar to human B27, and Papa-B similar to chimpanzee Patr-B*17:03^28,63^. Although de Groot et al.^28^ grouped all chimpanzee Patr-B*17 alleles together, we differentiated them into two groups, Patr-B*17:03 and Patr-B*17:01, based on differences in B and F anchor pocket composition and predicted P2 amino acid preferences (Supplementary Fig. 4).

### Laverania *LSA-1 ls6 peptide diversity*

The LSA-1 ls6 nonamer peptide (KPIVQYDNF, *P. falciparum* positions 1786-94)^11^ was used as a query to search PlasmoDB^37^ (https://plasmodb.org) to examine variation within the epitope (Fig. 4). Represented were *P. falciparum* (16 sequences), *P.* *praefalciparum* (1), *P.* *reichenowi* (2), *P.* *billcollinsi* (1),  *P.* *gaboni* (1), and *P. adleri* (1) (Supplementary Table 7). Since LSA-1 sequences were not available from *P. lomamiensis* and *P. blacklocki*, we used limited dilution PCR^14–16^ to generate *LSA-1* sequences from ape faecal samples known to contain these and other *Laverania* species (see Supplementary Methods). A 661 bp region containing the C-terminus was amplified, MiSeq sequenced and assembled using Geneious 2022.0.2 (https://www.geneious.com). In total, 22 new *Laverania* LSA-1 sequences were generated and deposited in GenBank (OM570838-OM570859).

### KIR epitope definition

Amino acid motifs at positions 76–83, encoded by exon 2, within MHC class I molecules are recognized by Killer-cell immunoglobulin-like receptors (KIR). For hominid MHC-B, two alternative KIR epitopes, Bw4 and C1, are carried by some of the allotypes^32,64^ (Fig. 3b, Fig. 2, Supplementary Table 9). Bw4 is defined primarily by an arginine at position 83, while C1 is defined primarily by a valine at position 76 and an asparagine at position 80. Among bonobos examined to date, these two epitopes are found exclusively among Papa-B, however these epitopes are also observed among other MHC-A or MHC-C class I allotypes in other extant hominids (i.e., humans, gorillas, chimpanzees, and orangutans)^32,64^.

### Statistical analyses

We tested for deviations between the observed genotype frequencies and those expected under Hardy-Weinberg equilibrium for TL2-W and TL2-E bonobos using GENEPOP v. 4.7.5 (Hardy-Weinberg exact test, using probability test). P values are estimated by GENEPOP using the Markov chain method^65,66^. Differences between the frequencies of the four *Papa-B* alleles shared by bonobos west and east of the Lomami River (Fig. 2) were tested using the Fisher’s exact test (two-tailed) in GraphPad QuickCalcs (graphpad.com/quickcalcs/contingency1 (accessed November 2022)).

To assess the association between particular KIR epitopes and peptide-binding supertypes among Papa-B allotypes, odds ratio statistics were calculated (MedCalc Software Ltd. Odds ratio calculator. https://www.medcalc.org/calc/odds_ratio.php (Version 20.215; accessed November 2022)) for all identified Papa-B (*N* = 37) as well as Papa-B only detected in wild populations (*N* = 29) (Fig. 2, Supplementary Table 9). Papa-B allotypes were categorized as either having the B07 peptide binding supertype or not (i.e. having either the B27 or Patr-B*17:03 supertype) and having a KIR epitope (either Bw4 or C1) or not.

We tested B07 supertype frequencies in TL2-W and TL2-E, both *Laverania*-infected, and in five other, non-*Laverania*-infected western bonobo sites, excluding sites with fewer than five sampled bonobos. Bonobo communities range from 13–48 individuals^67–69^, with five individuals representing at least 10% of a community. For each site, we grouped Papa-B allotypes into two categories: B07 or Other (B27 or Patr-B*17:03) (Fig. 2, Supplementary Table 3). We first compared the B07 frequencies among the five western populations, excluding TL2-W, using a chi-square test and did not detect a difference (χ^2^ = 1.319, df = 4, *p* = 0.86). Therefore, we proceeded by pooling those five populations and testing them as a single population (Other-West) compared to each of TL2-W and TL2-E, and we compared TL2-W to TL2-E, using the Fisher’s exact test (two-tailed, GraphPad QuickCalcs, graphpad.com/quickcalcs/contingency1/ (accessed November 2022)) and Bonferroni adjusted alpha levels of 0.0167 per test (0.05/3).

We also tested for significant differences between the absence of KIR epitopes in TL2-E and the frequencies of KIR epitopes among the six western bonobo populations, including TL2-W. For each population, we grouped Papa-B allotypes into two categories: KIR (either the Bw4 or C1 epitope) or None (Fig. 2, Supplementary Table 3). We first compared the B07 frequencies among the six western populations using a chi-square test. Because we detected a significant difference (χ^2^ = 23.478, df = 5, *p* = 0.0003), we compared the frequencies in each western population individually against TL2-E using the Fisher’s exact test (two-tailed, GraphPad QuickCalcs, graphpad.com/quickcalcs/contingency1/ (accessed November 2022)) and Bonferroni adjusted alpha levels of 0.0083 per test (0.05/6).

### Reporting summary

Further information on research design is available in the Nature Portfolio Reporting Summary linked to this article.

## Supplementary information

Supplementary Information

Reporting Summary

## Data Availability

New *Papa-B* sequences generated in this study have been deposited in GenBank under accession codes MW039484-MW039487 and ON936818-ON936819 (https://www.ncbi.nlm.nih.gov/genbank/) and were also deposited in the IPD-MHC database (https://www.ebi.ac.uk/ipd/mhc/group/NHP/). New bonobo mitochondrial DNA and LSA-1 sequences generated in this study have also been deposited in GenBank under accession codes ON936815-ON936817 and OM570838-OM570859, respectively. Previously identified LSA-1 sequences used in this study are available in PlasmoDB (https://plasmodb.org), and sequence IDs are provided in the Supplementary Information.  Source data are provided with this paper.

## Source data

Source Data
