# Supplementary material for: Malaria-driven adaptation of MHC class I in wild bonobo populations
Source: Nat Commun. 2023 Feb 23;14:1033. doi: 10.1038/s41467-023-36623-9 (PMC9950436; doi:10.1038/s41467-023-36623-9)
Supplement: Supplementary file 1 — Supplementary Information [file 41467_2023_36623_MOESM1_ESM.pdf]

Supplementary Information

Malaria-driven adaptation of MHC class I in wild bonobo populations

Emily E. Wroblewski[1*], Lisbeth A. Guethlein[2], Aaron G. Anderson[1], Weimin Liu[3], Yingying Li[3], Sara E. Heisel[1], Andrew Jesse Connell[3,4], Jean-Bosco N. Ndjango[5], Paco Bertolani[6], John A. Hart[7], Terese B. Hart[7], Crickette M. Sanz[1,8], David B. Morgan[9], Martine Peeters[10], Paul M. Sharp[11,12], Beatrice H. Hahn[3,4], and Peter Parham[2,13]

[1]Department of Anthropology, Washington University in St. Louis, Saint Louis, MO, 63130, USA
[2]Department of Structural Biology, Stanford University School of Medicine, Stanford, CA, 94305, USA
[3]Department of Medicine, Perelman School of Medicine, University of Pennsylvania, Philadelphia, PA, 19104, USA
[4]Department of Microbiology, Perelman School of Medicine, University of Pennsylvania, Philadelphia, PA, 19104, USA
[5]Department of Ecology and Management of Plant and Animal Resources, Faculty of Sciences, University of Kisangani, BP 2012 Kisangani, Democratic Republic of the Congo
[6]Institute of Human Sciences, School of Anthropology and Museum Ethnography, University of Oxford, Oxford, United Kingdom
[7]Frankfurt Zoological Society, Lomami National Park Project, Kinshasa, Democratic Republic of the Congo
[8]Congo Program, Wildlife Conservation Society, Brazzaville, Republic of Congo
[9]Lester E. Fisher Center for the Study and Conservation of Apes, Lincoln Park Zoo, Chicago, IL, 60614, USA
[10]Recherche Translationnelle Appliquée au VIH et aux Maladies Infectieuses, Institut de Recherche pour le Développement, University of Montpellier, INSERM, 34090 Montpellier, France
[11]Institute of Ecology and Evolution, University of Edinburgh, Edinburgh EH9 3FL, United Kingdom
[12]Centre for Immunity, Infection, and Evolution, University of Edinburgh, Edinburgh EH9 3FL, United Kingdom
[13]Department of Microbiology & Immunology, Stanford University School of Medicine, Stanford, CA, 94305, USA

Supplementary Table 1. Newly *Papa-B* genotyped bonobos at TL2 and BX

Population	Bonobo ID[a]	*Plasmodium* infection	*Papa-B*			
			Allele 1[b]	***Allele 2***[b]	**Number of faecal samples**	**Amplicons cloned**
**BX**	BX01		*15:01*	*15:01*	1	NGS[e]
**TL2-E**	TL01.1		*01:01/3*	*15:01*	1	X
	TL01.2		*09:02*	*15:01*	6	
	TL03		*01:01/3*	*15:01*	6	
	TL04	X	*01:01/3*	*09:02*	1	
	TL05	X	*09:02*	*15:01*	2	
	TL06	X	*15:01*	*19:01*	3	X
	TL07	X	*01:01/3*	*01/04/09*[c]	3	
	TL08		*15:01*	*Unknown*[d]	2	X
	TL09		*15:01*	*15:01*	2	X
	TL11		*01:01/3*	*15:01*	1	
	TL12		*15:01*	*Unknown*	1	
	TL13	X	*01:01/3*	*09:02*	1	
	TL14		*01:01/3*	***01:04***	1	X
	TL15	X	*09:02*	*15:01*	3	X
	TL16		*01:01/3*	*09:02*	1	X
	TL17		*15:01*	*19:01*	7	X
	TL18	X	*09:02*	*15:01*	3	
	TL20	X	***23:01***	*16:02*	2	X
	TL21		*15:01*	*15:01*	1	
	TL22		*09:02*	*15:01*	1	
	TL23		*01:01/3*	*04:01*	1	X
	TL24.1		***23:01***	*16:02*	1	X
	TL24.2	X	*01:01/3*	*19:01*	3	
	TL25	X	*15:01*	*Unknown*	1	
	TL26		*09:02*	*16:02*	6	X
	TL27	X	*15:01*	*16:02*	2	X
	TL28		*09:02*	*16:02*	3	
	TL29		*01:01/3*	*09:02*	2	X
	TL31		*01:01/3*	*09:02*	1	
	TL32		*09:02*	*16:02*	1	
	TL33	X	*01:01/3*	*15:01*	2	
	TL34		*15:01*	*15:01*	1	
	TL36		*01:01/3*	*15:01*	1	
	TL37	X	*01:01/3*	*15:01*	2	X
	TL38		*01:01/3*	*19:01*	1	
	TL39.1		*01:01/3*	*16:02*	1	X
	TL39.2	X	*01:01/3*	*15:01*	1	
	TL40.1		*01:01/3*	*01/04/09*	1	
	TL40.2		*04:01*	*16:02*	1	X
	TL41	X	*01:01/3*	*04:01*	2	
	TL43	X	*01:01/3*	***01:04***	2	X
	TL44		*09:02*	*09:02*	4	X
	TL45	X	*01:02*	*16:02*	2	
	TL46	X	*01:01/3*	*09:02*	1	
	TL47	X	*09:02*	*15:01*	1	
	TL48		*15:01*	*19:01*	1	
	TL49	X	*15:01*	*15:01*	3	
	TL51		*16:02*	*19:01*	1	X
	TL52		*15:01*	*Unknown*	1	X
	TL53		*15:01*	*16:02*	1	
	TL55		*04:01*	*15:01*	1	X
	TL56		*04:01*	*15:01*	1	X
**TL2-W**	TL57		*01:01/3*	*02:02*	3	X
	TL58		*07:01*	***04:02***	3	X
	TL59		*02:02*	*14:01*	1	X
	TL60	X	*02:02*	*14:01*	2	X
	TL61	X	*09:01*	*10:01*	4	X
	TL62		*02:02*	***24:01***	1	X
	TL63	X	*12:02*	***24:01***	1	X

[a]Each row represents an individual bonobo as identified by mitochondrial and microsatellite characterization[1-3], with the corresponding Papa-B genotype indicated. Extensions of 0.1 and 0.2 indicate individuals differentiated by *Papa-B* genotyping with otherwise identical STR genotypes.
[b]New *Papa-B* alleles are bolded.
[c]An exon 2 sequence was obtained, but an exon 3 sequence could not be confidently assigned to differentiate between *Papa-B*01:02*, *04:01*, and *09:02*, which share an exon 2 sequence.
[d]A second *Papa-B* allele could not be assigned.
[e]NGS indicates that the genotype for the BX sample was confirmed using the NGS methodology.

Supplementary Table 2. B07 phenotype distribution within each study population

	Population	# bonobos with complete genotypes[a]	Bonobos with B07 phenotype	B07 Phenotype[b] 2 Papa-B	1 Papa-B	None
West of Lomami	ML	14	Count	0	2	12
			Frequency	0	0.1429	0.8571
	LK	17	Count	0	6	11
			Frequency	0	0.3529	0.6471
	IK	18	Count	0	6	12
			Frequency	0	0.3333	0.6667
	BN	22	Count	0	9	13
			Frequency	0	0.4091	0.5909
	KR	36*	Count	1	8	27
			Frequency	0.0278	0.2222	0.7500
	TL2-W	7	Count	1	5	1
			Frequency	0.1429	0.7143	0.1429
East of Lomami	TL2-E	46*	Count	30	15	1
			Frequency	0.5769	0.2885	0.0192
	TS	1*	Count	1	0	0
			Frequency	NA	NA	NA
	BX	1	Count	1	0	0
			Frequency	NA	NA	NA
	BJ	2	Count	2	0	0
			Frequency	NA	NA	NA
	LY	1	Count	1	0	0
			Frequency	NA	NA	NA
	LI	1	Count	1	0	0
			Frequency	NA	NA	NA

[a]Asterisks indicate where individuals with second alleles designated as “Unknown” were excluded.
[b]Source data for newly genotyped populations (TL2-W, TL2-E, TS, BX, LY, LI) are provided in Supplementary Tables 1 and 4. Source data for the other populations were reported previously[4].

Supplementary Table 3. Numbers of B07 supertype and KIR epitope positive and negative Papa-B within each study population

Population	Papa-B count[b]	
	Supertype	
	B07	Other
ML	6	22
LK	6	28
IK	6	30
BN	9	35
KR	10	63
Other-West[a]	37	178
TL2-W	7	7
TL2-E	81	17
	KIR epitope	
Population	None	Epitope
ML	2	26
LK	8	26
IK	17	19
BN	23	21
KR	36	37
TL2-W	4	10
TL2-E	100	0

[a]For supertype comparisons, the five populations west of the Lomami, excluding TL2-W, were combined as "Other-West".
[b]Source data are available in Table 1.

Supplementary Table 4. Newly genotyped bonobo fecal samples from bonobo field sites LI, TS, and LY

Population (ID)	Fecal sample code	Bonobo ID	*Plasmodium* infection	mtDloop haplotype (Genbank accession)	Sex (AMEL)[a]			Final *Papa-B* genotype[b]		*Papa-B* NGS (all samples)[c,e]				*Papa-B* Sanger (select samples)[d,e]			
						Allele 1	*Allele 2*	*Allele 1*	*Allele 2*	*E2 Allele 1*	*E2 Allele 2*	*E3 Allele 1*	*E3 Allele 2*	*E2 Allele 1*	*E2 Allele 2*	*E3 Allele 1*	*E3 Allele 2*
**Lokoli forest (LI)**	**LI5125**	LI1	neg	ON936815	M	212-a	218-b	*15:01*	*15:01*	*15:01*		*15:01/2*					
**Tshuapa-Lomami (TS)**	**TS5137**	TS1	neg	ON936816	M	212-a	218-b	*09:02*	***23:02***	*01:01/3*	*01:02/04:01/09:02*	*15:01/2*			*01:02/04:01/09:02*		*09:01/2*
	**TS5138**	TS2	neg	ON936817	M	212-a	218-b	*01:01/3*	*U*	*01:01/3*		*01:01/01:03/01:06*		*01:01/3*			
**Lobaye forest (LY)**	**LY5308**	LY1	neg	ON936816	M	212-a	218-b	*04:01*	***15:03***	*01:02/04:01/09:02*	*15:01*		***15:03***			*04:01*	***15:03***
	**LY5309**		neg	ON936816	M	212-a	218-b	*04:01 (e3)*	***15:03***		*15:01*	*04:01*	***15:03***				
	**LY5310**		neg	ON936816	M	212-a	218-b	*04:01*	***15:03***		*15:01*	*04:01*		*01:02/04:01/09:02*	*15:01*	*04:01*	***15:03***
	**LY5311**		neg	ON936816	M	212-a	218-b	*04:01*	***15:03***	*01:02/04:01/09:02*		*04:01*				*04:01*	***15:03***
	**LY5312**		neg	ON936816	M	212-a	218-b	*04:01*	***15:03***	*01:02/04:01/09:02*	*15:01*	*04:01*	***15:03***				
	**LY5313**		neg	ON936816	M	212-a	218-b	*04:01*	***15:03***	*01:02/04:01/09:02*	*15:01*	*04:01*	***15:03***				
	**LY5314**		neg	ON936816	M	212-a	218-b	*04:01*	***15:03***	*01:02/04:01/09:02*				*01:02/04:01/09:02*	*15:01*	*04:01*	***15:03***
	**LY5315**		neg	ON936816	M	212-a	218-b	*04:01*	***15:03***	*01:02/04:01/09:02*	*15:01*		***15:03***	*01:02/04:01/09:02*	*15:01*	*04:01*	***15:03***
	**LY5316**		neg	ON936816	M	212-a	218-b	*04:01*	***15:03***	*01:02/04:01/09:02*	*15:01*		***15:03***	*01:02/04:01/09:02*	*15:01*	*04:01*	***15:03***
	**LY5317**		neg	ON936816	M	212-a	218-b	*04:01*	***15:03***	*01:02/04:01/09:02*				*01:02/04:01/09:02*	*15:01*	*04:01*	***15:03***
	**LY5318**		neg	ON936816	M	212-a	218-b	*04:01*	***15:03***	*01:02/04:01/09:02*	*15:01*	*04:01*	***15:03***				
	**LY5319**		neg	ON936816	M	212-a	218-b	*04:01*	***15:03 (e3)***	*01:02/04:01/09:02*		*04:01*	***15:03***				
	**LY5320**		neg	ON936816	M	212-a	218-b	*04:01*	***15:03 (e3)***	*01:02/04:01/09:02*		*04:01*	***15:03***				
	**LY5321**		neg	ON936816	M	212-a	218-b	*04:01*	***15:03***	*01:02/04:01/09:02*	*15:01*	*04:01*	***15:03***				
	**LY5322**		neg	ON936816	M	212-a	218-b	*04:01*	***15:03***	*01:02/04:01/09:02*	*15:01*	*04:01*	***15:03***				
	**LY5323**		neg	ON936816	M	212-a	218-b	*04:01*	***15:03***	*01:02/04:01/09:02*	*15:01*		***15:03***			*04:01*	***15:03***
	**LY5324**		neg	ON936816	M	212-a	218-b	*04:01*	***15:03***	*01:02/04:01/09:02*	*15:01*	*04:01*	***15:03***	*01:02/04:01/09:02*	*15:01*	*04:01*	***15:03***

Fecal sample code	D18s536		D4s243		D10s676		D9s922		D2s1326		D2s1333		D4s1627		D9s905		D5s1457		D1s550		D4s2408		D11s2002		D17s974	
	Allele 1	*Allele 2*	*Allele 1*	*Allele 2*	*Allele 1*	*Allele 2*	*Allele 1*	*Allele 2*	*Allele 1*	*Allele 2*	*Allele 1*	*Allele 2*	*Allele 1*	*Allele 2*	*Allele 1*	*Allele 2*	*Allele 1*	*Allele 2*	*Allele 1*	*Allele 2*	*Allele 1*	*Allele 2*	*Allele 1*	*Allele 2*	*Allele 1*	*Allele 2*
**LI5125**	153-a	153-a	196-a	204-a	177-b	189-a	301-e	309-a	254-c	254-c			194-a	214-a			131-c	131-c	158-b	162-a	284-a	288-a	166-b	174-a		
**TS5137**	141-a	157-a			173-a	173-a			250-b	262-c	294-a	294-a	194-a	210-b			131-c	135-b			284-a		166-b	166-b		188-a
**TS5138**	153-a	157-a			173-a	173-a	301-f	301-f	262-c	270-b			190-a	210-b	282-b	282-b	163-a	163-a	147-a	158-a	284-a	288-a	162-a	174-b	184-a	184-a
**LY5308**	141-a	153-a			169-a	173-a	297-d		238-a	238-a	294-a	294-a	190-a	210-b		282-b	115-b		158-a	158-a	284-a		166-a	166-b	184-b	188-a
**LY5309**	141-a	153-a	196-a	200-a	169-a	173-a		305-c	238-a	238-a			190-a	210-b	274-a		115-b	119-a	158-a	158-a	284-a	288-a	166-a	166-b	184-b	188-a
**LY5310**	141-a	153-a			169-a	173-a	297-d	305-c	238-a	262-c	294-a	294-a	190-a	210-b			115-b	119-a	158-a	158-a			166-a	166-b	184-b	188-a
**LY5311**	141-a	153-a			169-a	173-a											115-b	119-a	158-a	158-a			166-a	166-b	184-b	188-a
**LY5312**	141-a	153-a	196-a		169-a	173-a			238-a	262-c			190-a	210-b			115-b	119-a	158-a	158-a		288-a	166-a	166-b		188-a
**LY5313**	141-a	153-a	196-a	200-a	169-a	173-a	297-d	305-c	238-a	262-c	294-a	294-a	190-a	210-b	274-a	282-b	115-b	119-a	158-a	158-a	284-a	288-a	166-a	166-b	184-b	188-a
**LY5314**	141-a	153-a	196-a		169-a	173-a	297-d	305-c	238-a	262-c	294-a	294-a	190-a	210-b		282-b	115-b	119-a	158-a	158-a	284-a	288-a	166-a	166-b	184-b	188-a
**LY5315**	141-a	153-a	196-a	200-a	169-a	173-a	297-d	305-c	238-a	262-c	294-a	294-a	190-a	210-b			115-b	119-a	158-a	158-a			166-a	166-b	184-b	188-a
**LY5316**	141-a	153-a	196-a		169-a	173-a	297-d	305-c	238-a	262-c	294-a	294-a	190-a	210-b	274-a	282-b	115-b	119-a	158-a	158-a	284-a	288-a	166-a	166-b	184-b	188-a
**LY5317**	141-a	153-a					297-d						190-a	190-a					158-a	158-a			166-a	166-b	184-b	188-a
**LY5318**	141-a						297-d	305-c											158-a	158-a						
**LY5319**	141-a	153-a			169-a	173-a												119-a	158-a	158-a			166-a	166-b	184-b	
**LY5320**	141-a	153-a			169-a	173-a	297-d	305-c	238-a	262-c	294-a	294-a	190-a	210-b	274-a	282-b	115-b	119-a	158-a	158-a		288-a	166-a	166-b	184-b	
**LY5321**	141-a	153-a	196-a	200-a	169-a	173-a	297-d	305-c	238-a	262-c			190-a	190-a			115-b	119-a	158-a	158-a	284-a	288-a	166-a	166-b		188-a
**LY5322**	141-a	153-a	196-a	200-a	169-a	173-a	297-d	305-c			294-a	294-a	190-a	190-a				119-a	158-a	158-a		288-a	166-a	166-b	184-b	
**LY5323**	141-a	153-a	196-a	200-a	169-a	173-a	297-d											119-a	158-a	158-a			166-a	166-b	184-b	
**LY5324**	141-a	153-a		200-a	169-a	173-a	297-d		238-a	262-c	294-a	294-a	190-a	210-b	274-a	282-b			158-a	158-a	284-a	288-a	166-a	166-b		188-a

[a]All individuals among these samples are heterozygous at their amelogenin gene, indicating that they are male[5].

[b]The final *Papa-B* genotype is based on both exon 2 (E2) and exon 3 (E3). In the final *Papa-B* genotype "(e3)" denotes when an allele was only detected by its exon 3 sequence. New *Papa-B* alleles are listed in bold. *Papa-B*23:02* is a recombinant between *Papa-B*01:01/3* (exon 2) and *Papa-B*15:01* (exon 3). *Papa-B*15:03* is identical to *B*15:01* but with 1 SNP in exon 3.

[c]Blank cells means an allele was not detected.

[d]Targeted PCR repeats and Sanger sequencing was done to confirm NGS genotypes. A blank cell in a Sanger column indicates that repeat PCRs were not done.

[e]Multiple alleles separated by a backslash (e.g., *01:02/04:01/09:02*) indicate that these alleles have the same exon sequence.

Supplementary Table 5. Frequency differences for the four *Papa-B* alleles shared between bonobos west and east of the Lomami River

***Papa-B* allele**	**p-value[a]**
09:01	0.1014
15:01	**<0.0001**
01:01/3	**<0.0001**
19:01	1.0

[a]p-values of two-tailed Fisher's Exact tests are shown (frequencies are given in Table 2).

Supplementary Table 6. Mitochondrial haplotype distribution among wild bonobos

GenBank Accession #	Haplotype[a]	West of Lomami						East of Lomami					
		ML	LK	IK	BN	KR	TL2-W	TL2-E	TS	BX	BJ	LY	LI
JQ866274	IK2876			x	x	x							
JQ866275	IK2879			x	x								
JQ866276	IK2885			x	x								
JQ866279	KR10			x	x	x							
JQ866278	KR3			x	x	x							
JQ866282	KR5			x	x	x							
JQ866284	KR6			x	x	x							
JQ866277	KR2					x							
JQ866280	KR23			x		x							
JQ866281	KR48					x							
JQ866283	KR66					x							
JQ866285	KR73					x							
JQ866292	LK664		x			x							
JQ866286	LK645		x										
JQ866287	LK646	x	x										
JQ866288	LK647	x	x										
JQ866289	LK648		x										
JQ866290	LK650		x										
JQ866291	LK652		x										
JQ866293	LK673	x	x										
JQ866294	ML5487	x											
JQ866295	ML5490	x											
JQ866296	ML5505	x											
KY790561	TL3924						x						
KY790562	TL3926						x						
KY790563	TL3931						x						
KY790564	TL3940						x						
KY790556	TL3846							x					
KY790552	TL3798‡							x	x			x	
**ON936816**	**TS5137‡**								x			x	
KY780555	TL3821							x					
KY790550	TL3795							x					
KY790551	TL3796							x					
KY790553	TL3803							x					
KY790554	TL3814							x					
KY790557	TL3876							x					
KY790558	TL3878							x					
KY790559	TL3883							x					
KY790560	TL3886							x					
JQ866273	BJ688							x			x		
KY790543	BX4799*									x			x
**ON936815**	**LI5125***												x
**ON936817**	**TS5138**								x				

[a]LI5125 and TS5137 are identical to, but 19 bp longer than BX4799 (asterisks) and TL3798 (double crosses), respectively.

Supplementary Table 7. LSA-1 ls6 epitope variation among *Laverania*

Host	*Laverania* species	Sequence ID[a]	GenBank	1	2[c]	3	4	5	6	7	8	9
Human	*P. falciparum*	**PF3D7 1036400**		**K**	**P**	**I**	**V**	**Q**	**Y**	**D**	**N**	**F**
		Pf7G8 100040500		.	.	.	.	.	.	.	.	.
		PfDd2 100041400		.	.	.	.	.	.	.	.	.
		PfGA01 100041400		.	.	.	.	.	.	.	.	.
		PfGB4 100041000		.	.	.	.	.	.	.	.	.
		PfGN01 100041600		.	.	.	.	.	.	.	.	.
		PfHB3 100040500		.	.	.	.	.	.	.	.	.
		PfIT 100040100		.	.	.	.	.	.	.	.	.
		PfKE01 100041400		.	.	.	.	.	.	.	.	.
		PfKH01 100040600		.	.	.	.	.	.	.	.	.
		PfKH02 100041500		.	.	.	.	.	.	.	.	.
		PfML01 100040200		.	.	.	.	.	.	.	.	.
		PfSD01 100040700		.	.	.	.	.	.	.	.	.
		PfSN01 100041600		.	.	.	.	.	.	.	.	.
		PfTG01 100041300		.	.	.	.	.	.	.	.	.
W. gorilla	*P. praefalciparum*	GTggg118 LSA1 SGA2.3	OM570859	.	.	.	.	.	.	.	.	.
Chimpanzee	*P. reichenowi*	PrCDC 1035500.1		.	.	.	.	.	.	.	.	.
		PrG01 1034700-t36 1		.	.	.	.	.	.	.	.	.
		SYpte57 LSA1 SGA10.1	OM570845	.	.	.	.	.	.	.	.	.
		SYptt45 LSA1 SGA10.2	OM570847	.	.	.	.	.	.	.	.	.
		SYptt45 LSA1 SGA10.5	OM570846	.	.	.	.	.	.	.	.	.
Bonobo	*P. lomamiensis*	TL2pp3862 LSA1 SGA2.5	OM570850	.	.	.	.	.	.	**N**	.	.
		TL2pp3912 LSA1 SGA2.8	OM570851	.	.	.	.	.	.	**N**	.	.
		TL2pp3912 LSA1 SGA2.3	OM570852	.	.	.	.	.	.	**N**	.	.
		KRpp10 LSA1 SGA2.1	OM570853	.	.	.	.	.	.	**N**	.	.
Chimpanzee	*P. billcollinsi*	PbilcG01 1035100-t36 1		**N**	.	.	**M**	.	.	.	.	.
		SYptt79 LSA1 SGA10.4	OM570839	**N**	.	.	**M**	.	.	.	.	.
		SYptt20 LSA1 SGA10.1	OM570848	**N**	.	.	**M**	.	.	.	.	.
W. gorilla	*P. blacklocki*	DDggg3567 LSA1 SGA2.4	OM570854	.	.	.	**I**	.	.	.	.	.
		DDggg4331 LSA1 SGA2.5	OM570855	.	.	.	**I**	.	.	.	.	.
		GTggg833 LSA1 SGA2.2[b]	OM570857	.	.	.	**I**	.	.	.	.	.
		GTggg119 LSA1 SGA2.2	OM570858	.	.	.	**I**	.	.	.	.	.
Chimpanzee	*P. gaboni*	PgabG01 1034400-t36 1		.	.	.	**M**	.	.	**E**	.	.
		SYptt63 LSA1 SGA10.3	OM570843	.	.	.	**M**	.	.	**E**	.	.
		SYptt92 LSA1 SGA10.8	OM570840	.	.	.	**M**	.	.	**E**	.	.
		SYpte70 LSA1 SGA10.2	OM570841	.	.	.	**M**	.	.	**E**	.	.
		SYptt82 LSA1 SGA10.1	OM570838	.	.	.	**M**	.	.	**E**	.	.
		SYpte37 LSA1 SGA10.1	OM570844	.	.	.	**M**	.	.	**E**	.	.
		SYptt63 LSA1 SGA10.2	OM570842	.	.	.	**M**	.	.	**E**	.	.
		SYptt13 LSA1 SGA10.1	OM570849	.	.	.	**M**	.	.	**E**	.	.
W. gorilla	*P. adleri*	Padl01 1035000-t36 1		.	.	.	**M**	.	.	**E**	.	.
		GTggg833 LSA1 SGA2.4[b]	OM570856	.	.	.	**M**	.	.	**E**	.	.

[a]Sequences obtained from PlasmoDB[6] are named by first giving a *Plasmodium* species abbreviation (*P. falciparum* (Pf), *P. reichenowi* (Pr), *P. billcollinsi* (Pbilc), *P. gaboni* (Pg), *P. adleri* (Padl)) and strain and a database sequence identification number. Sequences obtained by SGA are named by the site code (TL2 (Tshuapa-Lomami-Lualaba, D.R.C.), KR (Kokolopori, D.R.C.), GT (Goualougo Triangle, Rep. of Congo), DD (Deng Deng, Cameroon), SY (Sanaga Yong Rescue Centre, Cameroon)), then an ape species origin abbreviation (bonobo, *Pan paniscus* (pp); Western lowland gorilla, *Gorilla gorilla gorilla* (ggg); central chimpanzee, *Pan troglodytes troglodytes* (ptt); Nigeria-Cameroon chimpanzee, *P. t. ellioti* (pte)), the sample number, and then LSA1 followed by an SGA sequence identification number (e.g. SGA2.5).

[b]The two sequences from the Western gorilla sample 833 are from different *Laverania* species, reflecting coinfection.

[c]The C-terminus portion of LSA-1, positions 1630-1909 (280 amino acids), contains the nonamer ls6 peptide (positions 1786-1794, corresponding to positions 1-9 in the peptide). Positions 2 and 9 (underlined) are the conserved anchor positions that hold the peptide within the MHC-B peptide-binding groove.

Supplementary Table 8. Odds ratio statistics testing the association between Papa-B KIR epitopes and peptide-binding

	Supertype	KIR	None	Statistics
**All Papa-B[a]**	B07<br>Other	7<br>7	13<br>11	Odds ratio: 1.1818<br>95% CI: 0.3157, 4.4239<br>z statistic: 0.248<br>Significance level: 0.8041
**30 wild Papa-B**	B07<br>Other	2<br>6	11<br>11	Odds ratio: 0.3333<br>95% CI: 0.0548, 2.2075<br>z statistic: 1.193<br>Significance level: 0.233

[a]All identified Papa-B (including the 30 wild Papa-B) are given in Supplementary Table 9.

Supplementary Table 9. All identified Papa-B allotypes and their associated KIR epitope and peptide-binding supertype

Papa-B[a]	Wild[d]	KIR	Supertype
*01:01	x		B07
*01:02	x		B07
*01:03			B07
*02:01		Bw4	B07
*02:02	x	Bw4	B07
*03:01		Bw4	B27
*04:01	x		B07
*05:01		Bw4	B07
*06:01		C1	B07
*07:01 (2)[b]	x	Bw4	B27
*08:01	x		B07
*08:02	x		B27
*09:01	x	C1	B07
*09:02	x		B07
*10:01	x		B27
*11:01	x		B27
*12:01	x	C1	B27
*12:02	x		B27
*13:01	x	Bw4	B27
*14:01	x	Bw4	Patr-B*17:03
*15:01	x		B07
*15:02			B07
*16:01	x		B27
*16:02	x		B27
*17:01	x		Patr-B*17:03
*17:02	x		Patr-B*17:03
*18:01	x		B27
*19:01	x		B27
*19:02	x		B27
*20:01	x	C1	B27
*21:01		Bw4	B07
*22:01		Bw4	B07
***24:01**	x	Bw4	B27
***04:02**	x		B07
***23:01**	x		B07
***01:04**	x		B07
***15:03**	x		B07
***23:02**	x		B07
*KR1e3[c]	x	Unknown	Unknown

[a]Listed are all identified Papa-B (33 Papa-B in the IPD-MHC database[7] as of July 2022 as well as the six new allotypes, in bold, found in TL2, TS, and LY bonobos.

[b]"(2)" denotes that Papa-B*07:01:01 and *07:01:02 are functionally identical (a single, synonymous mutation in exon 1 differentiates them).
[c]KR1e3 was not analysed in the Odds Ratio statistics (Supplementary Table 8) because it is only characterized for exon 3, and, therefore, its KIR epitope and supertype are unknown.
[d]An "x" indicates alleles that were detected in wild populations (N=31) (Table 2).

Supplementary Table 10. Locus-specific primer sequences for bonobo genotyping

	Locus	Forward primer sequence (5' - 3')	Reverse primer sequence (5' - 3')
MHC-B	exon 2	B2-425For: ATGGCCTCTGCCGGGAGGAG	B2-425Rev: CGTSGGGGWTGGGGAGTCGT
	exon 2, *B*17*[a]	(B2-425For)	B2-429Rev: GGGTTGGGGAGGGGTCGTGA
	exon 3	B3-411For: GGCCAAAATCCCCGCGGGTT	B3-411Rev: GTGGGAGGCCATCCCSGGC
**Microsatellite**	D18S536	ATTATCACTGGTGTTAGTCCTCTG	CACAGTTGTGTGAGCCAGTC
	D4S243	TCAGTCTCTCTTTCTCCTTGCA	TAGGAGCCTGTGGTCCTGTT
	D10S676	GAGAACAGACCCCCAAATCT	ATTTCAGTTTTACTATGTGCATGC
	D9S922	TCAGAGGACCACTGCCTAAG	CTGATGGGATTTGTGCCTAT
	D2S1326	AGACAGTCAAGAATAACTGCCC	CTGTGGCTCAAAAGCTGAAT
	D2S1333	CTTTGTCTCCCCAGTTGCTA	TCTGTCATAAACCGTCTGCA
	D4S1627	AGCATTAGCATTTGTCCTGG	GACTAACCTGACTCCCCCTC
	D9S905	GTGGGAAAATTGGCCTAAGT	CTTCTGAGCCTCACACCTGT
	D5s1457	TAGGTTCTGGGCATGTCTGT	TGCTTGGCACACTTCAGG
	D1s550	CCTGTTGCCACCTACAAAAG	TAAGTTAGTTCAAATTCATCAGTGC
	D4s2408	AATAAACTTCAACTTCAATTCATCC	AGGTAAAGGCTCTTCTTGGC
	D11s2002	CATGGCCCTTCTTTTCATAG	AGTGTGAGCCACCACACCAGC
	D17S974	AGACCCTGTCTCAGATAGATGG	TAAAATAGAAAGTGCCCCTCC
**Sex**	amelogenin	ACCTCATCCTGGGCACCCTGG	AGGCTTGAGGCCAACCATCAG
**Mitochondrial**	D-loop	L15997: CACCATTAGCACCCAAAGCT	H16498: CCTGAAGTAGGAACCAGATG

[a]Specific primer combination to amplify exon 2 of Patr-B*17 lineage alleles.

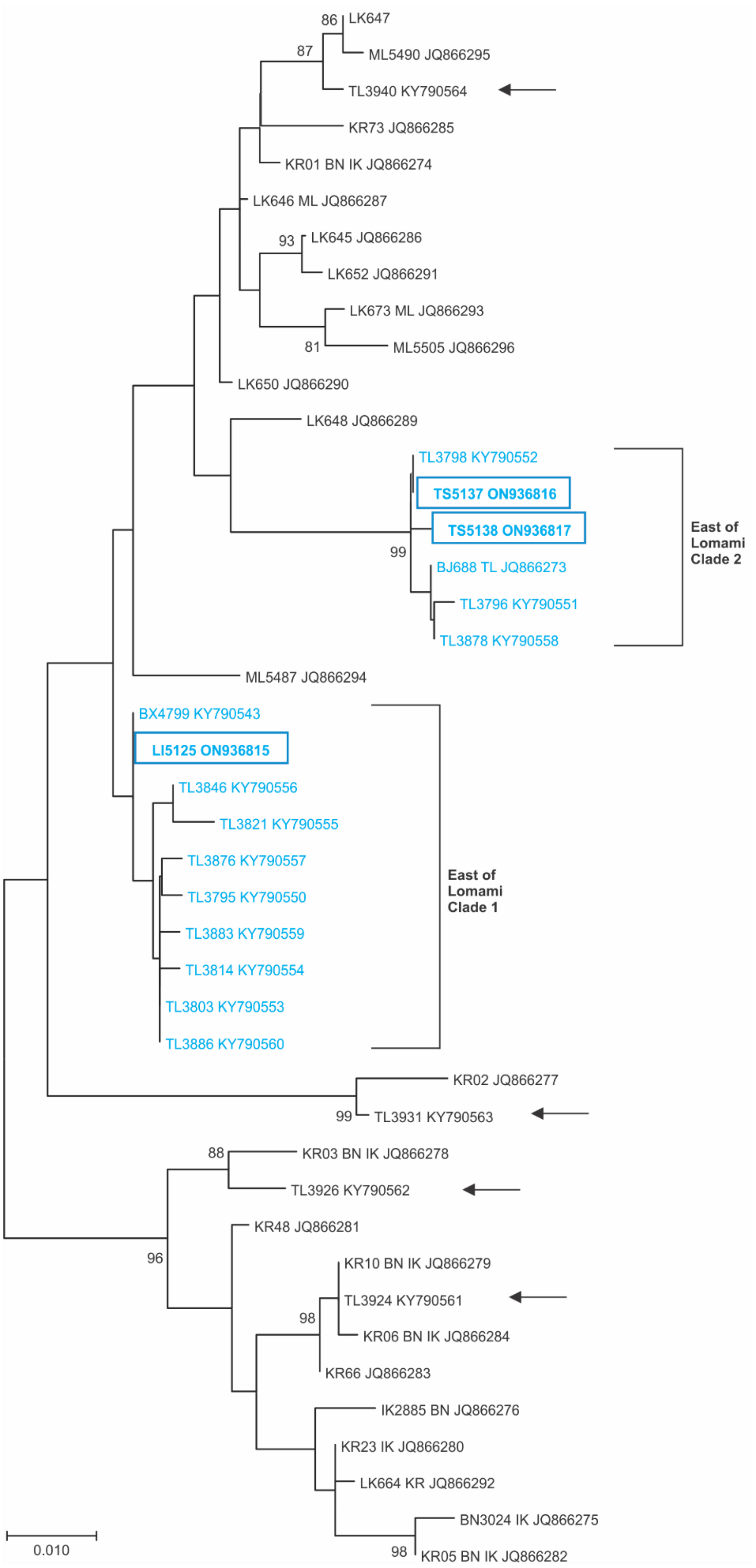
86
LK647
87
ML5490 JQ866295
TL3940 KY790564
KR73 JQ866285
KR01 BN IK JQ866274
LK646 ML JQ866287
93
LK645 JQ866286
LK652 JQ866291
LK673 ML JQ866293
81
ML5505 JQ866296
LK650 JQ866290
LK648 JQ866289
TL3798 KY790552
TS5137 ON936816
TS5138 ON936817
99
BJ688 TL JQ866273
TL3796 KY790551
TL3878 KY790558
East of Lomami Clade 2
ML5487 JQ866294
BX4799 KY790543
LI5125 ON936815
TL3846 KY790556
TL3821 KY790555
TL3876 KY790557
TL3795 KY790550
TL3883 KY790559
TL3814 KY790554
TL3803 KY790553
TL3886 KY790560
East of Lomami Clade 1
KR02 JQ866277
99
TL3931 KY790563
88
KR03 BN IK JQ866278
TL3926 KY790562
KR48 JQ866281
96
KR10 BN IK JQ866279
TL3924 KY790561
98
KR06 BN IK JQ866284
KR66 JQ866283
IK2885 BN JQ866276
KR23 IK JQ866280
LK664 KR JQ866292
BN3024 IK JQ866275
98
KR05 BN IK JQ866282
0.010

Supplementary Fig. 1 The Lomami River represents a barrier to bonobo gene flow. A maximum likelihood tree of previously reported and newly generated bonobo mitochondrial haplotypes with known sampling location is shown (haplotypes are listed in Supplementary Table 7). The tree was constructed in MEGA X[8] using the Maximum Likelihood method, with 10,000 replications, and the Hasegawa-Kishino-Yano (HKY) model[9] with a discrete Gamma (+G) distribution used to model evolutionary rate differences among sites. Bootstrap values are shown for branches with ≥80% support. Haplotypes are named by the two-letter site code for the population in which it was initially identified and an identification number, followed by the GenBank accession number. Haplotypes from bonobos East of the Lomami River (blue) form two clades. New haplotypes from bonobos sampled in TS, LY, and LI are bolded and boxed and cluster within Eastern clade 1 or Eastern clade 2, consistent with the Lomami River acting as a barrier to gene flow. Four haplotypes marked by arrows are those found in TL2-W. LI5125 and TS5137 are each identical to a previously identified sequence (BX4799 and TL3798, respectively), but are 19 bp longer (due to differences in the sequencing method).

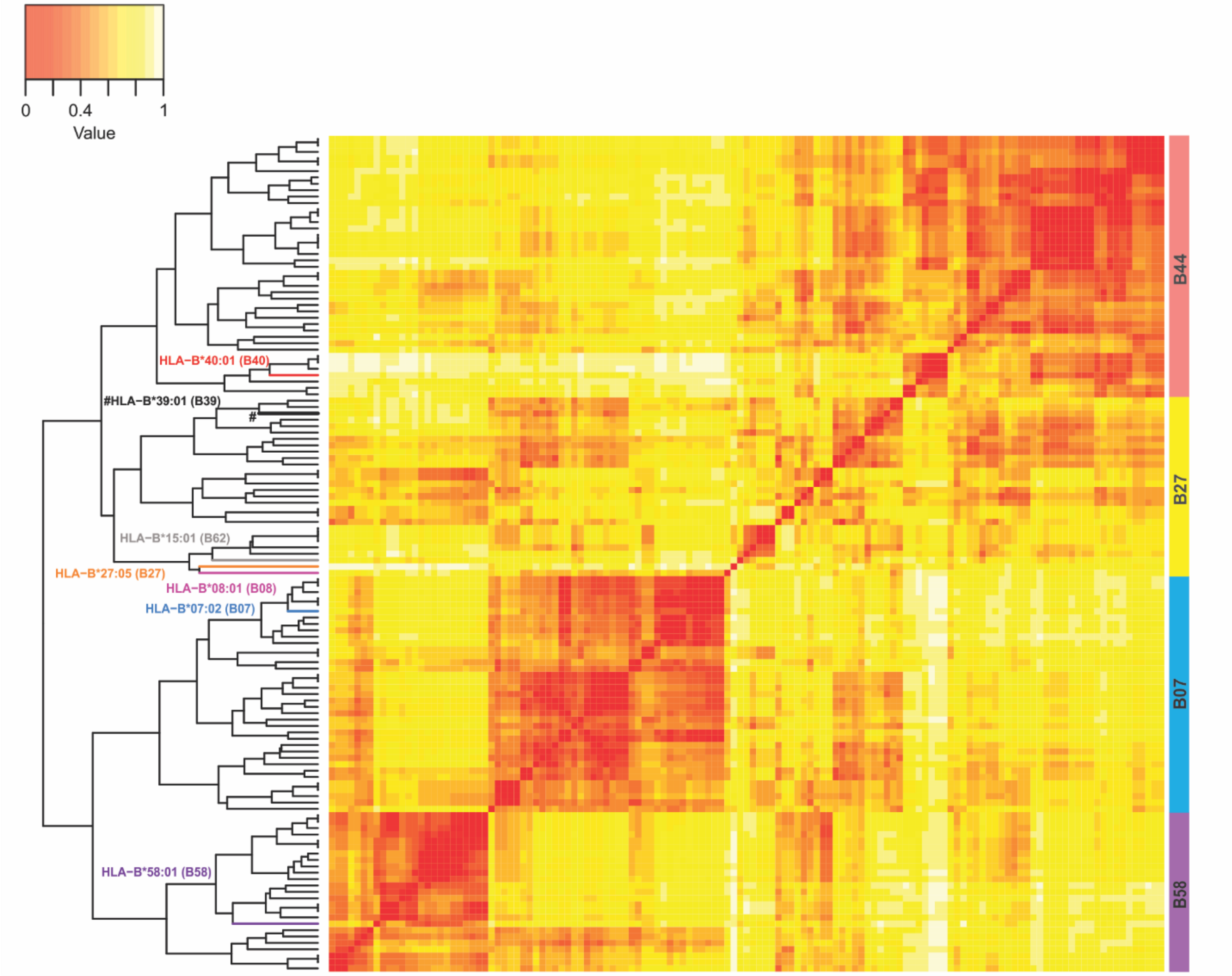

Supplementary Fig. 2 MHCcluster heatmap of predicted peptide binding similarity of bonobo, chimpanzee, and representative human MHC-B. Heatmap of all 39 Papa-B and 85 Patr-B allotypes, as well as select HLA-B allotypes representing different supertypes, indicating relative similarity in peptide binding profiles (as indicated by a color key). Papa-B and Patr-B allotypes are listed in Supplementary Fig. 4. The tree to the left illustrates the supertype clusters that are also marked by the coloured bars on the right. Human HLA-B allotypes are labeled and individually coloured (colours correspond with those used in Supplementary Fig. 4). HLA-B*39:01 (B39) is denoted by the hash mark and a thickened line.

Supertype	Bonobo	Chimpanzee
B07	0.513 (20)	0.188 (16)
B27	0.410 (16)	0.024 (2)
B44		0.271 (23)
B58		0.282 (24)
Patr-B*02:01		0.153 (13)
Patr-B*08:01		0.024 (2)
Patr-B*17:01		0.024 (2)
Patr-B*17:03	0.077 (3)	0.035 (3)

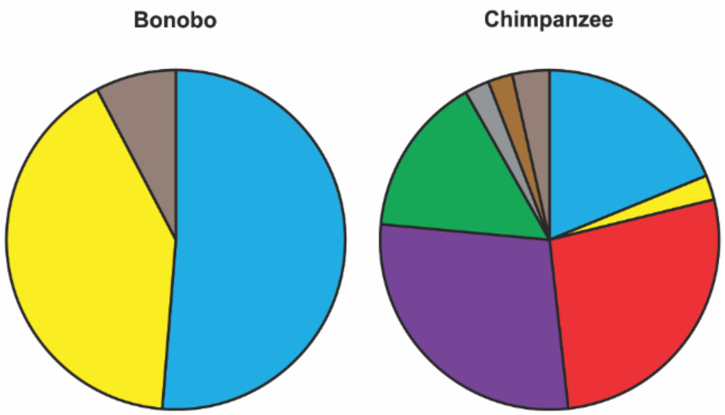

Supplementary Fig. 3 Supertype frequencies among 39 bonobo Papa-B and 85 chimpanzee Patr-B allotypes. Papa-B and Patr-B allotypes are listed in Supplementary Fig. 4. Supertype colours also correspond with those used in Supplementary Fig. 4. The number of MHC-B allotypes representing the supertype are in parentheses.

	MHCcluster predictions			Supertype			B POCKET											F POCKET										
	Enriched anchor residues		Prediction accuracy		Preferred		7	9	24	45	62	63	66	67	70	99	159	74	77	80	81	84	95	97	116	118	143	147
**MHC-B allotype**	**P2**	**P9**		**Group**	**P2**	**P9**	**Y**	**Y**	**S**	**E**	**R**	**N**	**I**	**S**	**N**	**Y**	**Y**	**D**	**S**	**N**	**L**	**Y**	**L**	**T**	**F**	**Y**	**T**	**W**
Patr-B*04:02:01:01	E,Q,D,M	L,I,V,A,F,M	0.814	**B44**	**E/Q/D**	**L/I**	-	-	T	K	-	E	-	-	-	F	-	Y	G	-	-	-	W	-	Y	-	-	-
Patr-B*04:01	E,Q,D,M	L,I,V,A,F,M	0.814				-	-	T	K	-	E	-	-	-	F	-	Y	G	-	-	-	W	-	Y	-	-	-
Patr-B*16:02	D,E,Q,M	I,L,V,A,F,M	0.903				-	-	T	K	-	E	-	-	-	F	-	-	-	-	-	-	W	-	Y	-	-	-
Patr-B*16:01:01	Q,E,D,M,R	L,I,V,A,M	0.870				-	-	T	K	-	E	-	-	-	F	-	-	-	-	-	-	W	-	Y	-	-	-
Patr-B*16:01:02	Q,E,D,M,R	L,I,V,A,M	0.870				-	-	T	K	-	E	-	-	-	F	-	-	-	-	-	-	W	-	Y	-	-	-
Patr-B*22:07:01:01	E,Q,D,M	L,I,V,A,M	0.823				-	-	T	K	-	E	-	-	-	F	-	Y	-	-	-	-	W	-	-	-	-	-
Patr-B*22:08	E,Q,D	L,I,F,V,M,W	0.852				-	-	T	K	-	E	-	-	-	F	-	Y	N	I	A	-	-	-	Y	-	-	-
Patr-B*22:02	E,Q,D	L,I,F,V,M,W	0.855				-	-	T	K	-	E	-	-	-	F	-	Y	N	I	A	-	-	-	Y	-	-	-
Patr-B*30:01	E,Q,D	L,I,V,F,M,W	0.798				-	-	T	K	-	E	-	-	-	-	-	Y	N	I	A	-	I	R	Y	-	-	-
Patr-B*23:07:01:01	E,D,Q,M	I,L,F,V,W	0.819				-	-	T	K	-	E	-	-	-	F	-	Y	N	I	A	-	-	-	-	-	-	-
Patr-B*34:01	E,Q,D	I,L,V,M	0.825				-	-	T	K	-	E	-	-	-	-	-	Y	N	I	A	-	W	-	Y	-	-	-
Patr-B*22:06:01:01	E,Q,D,K,M	L,I,M,V,F	0.890				-	-	T	K	-	E	-	-	-	F	-	Y	-	-	-	-	-	-	Y	-	-	-
Patr-B*22:01:01:01	E,Q,D,K,M	L,I,M,V,F	0.890				-	-	T	K	-	E	-	-	-	F	-	Y	-	-	-	-	-	-	Y	-	-	-
Patr-B*22:04:01:01	E,Q,D,M	L,I,M,V,F	0.887				-	-	T	K	-	E	-	-	-	F	-	Y	-	-	-	-	-	-	Y	-	-	-
Patr-B*22:03:01:01	Q,E,D,K,M	L,F,I,M	0.859				-	-	T	K	-	E	-	-	-	F	-	Y	-	-	-	-	-	-	H	-	-	-
Patr-B*05:01:01:01	Q,E,K,H,M	L,I,V,M,F	0.850		**K,Q**	**L**	-	-	-	-	-	E	-	-	-	-	-	Y	-	-	-	-	I	R	Y	-	-	-
Patr-B*02:03	Q,E,K,H,M	L,I,V,M,F	0.850		**Q (E)**	**L**	-	-	-	-	-	E	-	-	-	-	-	Y	-	-	-	-	I	R	Y	-	-	-
Patr-B*02:01	Q,E,K,H,M	L,I,V,M,F	0.850	**Patr-B*02:01**			-	-	-	-	-	E	-	-	-	-	-	Y	-	-	-	-	I	R	Y	-	-	-
Patr-B*05:02	Q,E,K,M,H	L,F,I,M,V	0.879				-	-	-	-	-	E	-	-	-	-	-	Y	-	-	-	-	I	R	Y	-	-	-
Patr-B*22:05:01:01	E,Q	L,F,I,M,V,Y	0.867		**E**	**L**	-	-	T	K	-	E	-	-	-	-	-	Y	-	-	-	-	-	R	H	-	-	-
Patr-B*39:01	E,Q,M	L,I,V,M,F	0.851				-	-	T	K	-	E	-	-	-	-	-	Y	-	-	-	-	I	R	Y	-	-	-
Patr-B*23:01:01	Q,E,S,A,M,D,H	I,L,V,F	0.760	**Patr-B*02:01**	**Q (E/A)**	**I**	-	-	-	-	-	E	-	-	-	-	-	Y	N	I	A	-	-	-	-	-	-	-
Patr-B*23:01:02:01	Q,E,S,A,M,D	I,L,V,F	0.760				-	-	-	-	-	E	-	-	-	-	-	Y	N	I	A	-	-	-	-	-	-	-
Patr-B*23:02:01:01	Q,A,E,G,K,S,M,H	I,L,F,W,M	0.775				-	-	-	-	-	E	-	-	-	-	-	Y	N	I	A	-	-	-	-	-	-	-
Patr-B*23:05:01:01	Q,E,G,D,A,M,H	I,F,L,W	0.781				-	-	-	-	-	E	-	-	-	-	-	Y	N	I	A	-	-	-	-	-	-	-
**Papa-B*24:01**	Q,E,M,D	I,L,F,V,W,M	0.653	**B27**	**Q (E/S/A)**	**I/L**	-	D	T	M	-	E	-	-	-	-	-	-	-	I	A	-	-	R	-	-	-	-
**Papa-B*16:02**	Q,S,M,G,E,R,N,H	L,I,F,V,M	0.663				-	D	T	M	-	E	N	-	-	-	-	-	-	-	-	-	-	R	-	-	-	-
**Papa-B*16:01:01:01**	Q,A,E,S,R,M	L,F,I,M,V	0.687				-	D	T	M	-	E	N	-	-	-	-	-	-	-	-	-	-	R	-	-	-	-
Patr-B*38:02:01:01	Q,E,K,M,R	F,L,M,I,Y	0.847	**Patr-B*02:01**	**Q (E/A)**	**F/L**	-	-	-	-	-	E	-	-	-	S	-	-	-	-	-	-	-	R	S	-	-	-
Patr-B*23:03	Q,E,A,K,M,D,H	L,I,F,V,M	0.789				-	-	-	-	-	E	-	-	-	-	-	Y	N	T	-	-	-	-	Y	-	-	-
Patr-B*23:06:01:01	Q,A,E,K,M,H	L,I,F,M	0.811				-	-	-	-	-	E	-	-	-	-	-	Y	N	T	-	-	-	-	-	-	-	-
**Papa-B*12:01**	Q,R,E,D,M	I,V,L,A,M	0.763	**B27**	**Q (R/A)**	**I/L**	-	D	T	M	-	E	-	-	-	-	-	-	G	-	-	-	W	-	-	-	-	-
**Papa-B*12:02**	Q,A,R,S,G,M	L,I,V,A,M	0.697				-	D	T	M	-	E	N	-	-	-	-	-	-	-	-	-	W	-	-	-	-	-
Patr-B*38:01	Q,R,A,K,P,E,M	I,Y,A,F,L,V,M,W	0.810	**Patr-B*02:01**	**Q (R)**	**I**	-	-	-	-	-	E	-	-	-	-	-	-	-	-	-	-	W	-	S	-	-	-
Patr-B*40:01:01:01	E,D,Q	I,V,L,A,M	0.834		**E/D**	**I/V/L**	-	H	T	K	-	E	-	-	-	-	-	Y	-	-	-	-	W	-	Y	-	-	-
Patr-B*40:01:01:02	E,D,Q	I,V,L,A,M	0.834				-	H	T	K	-	E	-	-	-	-	-	Y	-	-	-	-	W	-	Y	-	-	-
Patr-B*40:02:01:01	E,D,Q	V,I,A,L,M	0.834				-	H	T	K	-	E	-	-	-	-	-	Y	-	-	-	-	W	-	Y	-	-	-
**HLA-B*40:01**	**E**	**L,I,V,F,M**	**1.000**	**B44**	**E**	**L**	**-**	**H**	**T**	**K**	**-**	**E**	**-**	**-**	**-**	**-**	**-**	**Y**	**-**	**-**	**-**	**-**	**-**	**R**	**Y**	**-**	**S**	**L**
Patr-B*30:02:01:01	E,D,Q	L,I,W,F,V,Y,M	0.844				-	-	T	K	-	E	-	-	-	-	-	Y	N	I	A	-	I	R	H	-	-	-
Patr-B*24:01:01:01	D,E,Q	I,V,L,A,M	**1.000**				-	-	T	K	-	E	-	-	-	F	-	-	N	T	-	-	W	-	-	-	-	-
Patr-B*24:02	D,E,Q	I,V,L,M	0.983				-	-	T	K	-	E	-	-	-	F	-	-	N	T	-	-	W	-	Y	-	-	-
**Papa-B*08:02**	R,A,Q,M,H	L,A,M,V,I,F	0.717	**B27**	**R/Q**	**L**	-	D	-	-	-	-	-	C	Q	-	-	-	-	-	-	-	-	-	-	-	-	-
**Papa-B*11:01**	R,P,A,Q,K,M,H	L,M,V,I,F	0.754				-	D	T	-	-	-	-	C	Q	-	-	-	-	-	-	-	-	-	-	-	-	-
**HLA-B*39:01**	**H,R,Q,K,P,M**	**L,I,V,M**	**1.000**	**[B39]**			-	-	-	-	-	-	-	C	-	-	-	-	-	-	-	-	-	R	-	-	-	-
**Papa-B*20:01**	R,Q,E,M,H,K	L,M,I,V,F	0.709				-	D	-	M	-	Q	-	-	-	-	-	-	G	-	-	-	-	R	-	-	-	-
**Papa-B*19:01:01:01**	Q,R,E,M	L,I,M	0.699				-	D	-	M	-	E	-	-	-	-	-	-	-	-	-	-	-	R	-	-	S	L
**Papa-B*19:02:01:01**	Q,R,L,M	L,F,M,I	0.726				-	D	-	M	-	E	-	-	-	-	-	-	-	-	-	-	-	R	S	-	S	L
Patr-B*21:02:01:01	Q,A,M,P,S	I,L,V,M,F	0.740	**Patr-B*17:03**	**Q (A)**	**I/L**	-	-	T	M	-	Q	-	-	-	-	-	Y	-	-	-	-	W	-	-	-	-	-
Patr-B*21:01:01:01	Q,A,L,M,S	L,I,F,M,V	0.789				-	-	T	M	-	Q	-	-	-	-	-	Y	-	-	-	-	I	-	-	-	-	-
Patr-B*23:04:01:01	Q,A,M,P	L,I,V,F,M	0.788	**Patr-B*02:01**	**Q (A)**	**L**	-	-	-	-	-	E	-	-	-	-	-	-	-	-	-	-	-	-	-	-	-	-
**Papa-B*17:02**	L,Q,M	L,I,V,F,M	0.702		**L**	**L**	-	F	T	M	-	Q	-	-	-	-	-	-	-	-	-	-	-	R	-	-	-	-
Patr-B*17:03	L,M,Q,R	L,V,I,M	0.677	**Patr-B*17:03**			-	F	T	V	-	Q	-	-	-	-	-	-	-	-	-	-	-	-	Y	-	S	F
Patr-B*03:01	S,T,A,Y,R,F	I,V,L,F,W	0.812	**B27**	**R***	**I,L**	-	D	T	M	-	E	N	V	S	F	-	-	N	I	A	-	W	-	Y	-	-	-
Patr-B*03:02:01:01	S,T,A,Y,R,F	I,V,L,F,W	0.812		**S**	**I**	-	D	T	M	-	E	N	V	S	F	-	-	N	I	A	-	W	-	Y	-	-	-
Patr-B*06:02:01:01	R,S,A,T,M	V,I,L,M	0.639	**B58**	**R**	**V**	-	D	-	M	-	E	N	V	S	-	-	-	G	-	-	-	W	-	Y	-	-	L
**Papa-B*22:01**	P,A,D,Q,E,S	I,L,V,F,W	0.769	**B07**	**P**	**I**	-	-	T	M	-	-	-	-	-	-	-	Y	N	I	A	-	W	-	-	-	-	-
**Papa-B*14:01:01:01**	L,Q,M,A,S	I,L,F,W	0.717	**Patr-B*17:03**	**L**	**I**	-	F	T	M	-	E	-	-	-	-	-	-	N	I	A	-	-	R	-	-	-	-
**Papa-B*13:01**	D,Q,R,A,M	I,F,L,V,W	0.713	**B27**	**D**	**I**	-	D	T	M	-	Q	-	-	-	F	-	-	N	I	A	-	W	-	H	-	-	-
Patr-B*08:01	L,S,A,M,Q,V,I,T	I,V,A,L	0.714	**Patr-B*08:01**	**L**	**I**	-	-	A	M	-	E	-	-	-	-	-	Y	G	-	-	-	W	-	-	-	-	-
Patr-B*08:02	L,S,A,M,Q,V,I,T	I,V,A,L	0.714				-	-	A	M	-	E	-	-	-	-	-	Y	G	-	-	-	W	-	-	-	-	-
**Papa-B*17:01:01:01**	L,S,M,T,A,Q	L,I,M,F	0.672	**Patr-B*17:03**	**L**	**L**	-	F	T	M	-	E	N	-	-	-	-	-	-	-	-	-	-	R	-	-	-	-
**Papa-B*07:01:01**	R,Q,H,P,Y,M	F,Y,W,L,I,M	0.728	**B27**	**R/Q**	**F/L**	-	D	T	-	-	-	-	C	Q	-	-	-	N	I	A	-	-	R	S	-	-	-
**Papa-B*07:01:02:01**	R,Q,H,P,Y,M	F,Y,W,L,I,M	0.728				-	D	T	-	-	-	-	C	Q	-	-	-	N	I	A	-	-	R	S	-	-	-
**Papa-B*03**	R,Q,H,P,Y,M	F,Y,W,L,I,M	0.728				-	D	T	-	-	-	-	C	Q	-	-	-	N	I	A	-	-	R	S	-	-	-
**Papa-B*10:01**	R,A,Q,H,M	L,F,Y,M,W	0.747				-	D	-	-	-	-	-	C	Q	F	-	Y	-	-	-	-	-	R	S	-	-	-
**Papa-B*18:01**	Q,L,R,M	F,Y,L,M,W	0.731				-	D	-	M	-	Q	-	-	-	-	-	-	-	-	-	-	-	K	D	-	-	-
**HLA-B*15:01**	**L,Q,M,I,S,V**	**F,Y,M**	**1.000**	B62	L	F	-	-	A	M	-	E	-	-	-	-	-	Y	-	-	-	-	-	R	S	-	-	-
**HLA-B*27:05**	**R**	**L,R,K,V,I,F,M**	**1.000**	B27	R	L	**-**	**H**	**T**	**-**	**-**	**E**	**-**	**C**	**K**	**-**	**-**	**-**	**D**	**T**	**-**	**-**	**-**	**N**	**D**	**-**	**-**	**-**
**HLA-B*08:01**	**L,A,P,M,I**	**L,V,I,A,F,M**	**1.000**	B08	L	L	-	D	-	-	-	-	-	F	-	-	-	-	-	-	-	-	-	S	Y	-	-	-

	MHCcluster predictions			Supertype			B POCKET											F POCKET										
	Enriched anchor residues		Prediction accuracy		Preferred		7	9	24	45	62	63	66	67	70	99	159	74	77	80	81	84	95	97	116	118	143	147
MHC-B allotype	P2	P9		Group	P2	P9	Y	Y	S	E	R	N	I	S	N	Y	Y	D	S	N	L	Y	L	T	F	Y	T	W
**Papa-B*01:04**	P,A	L,V,I,F,M,A	0.867		P	L/A/F/I	-	-	-	-	-	-	-	Y	Q	-	-	-	-	-	-	-	-	-	-	-	-	-
**Papa-B*01:02:01:01**	P,A	L,V,I,F,M,A	0.867				-	-	-	-	-	-	-	Y	Q	-	-	-	-	-	-	-	-	-	-	-	-	-
Patr-B*13:01:01:01	P	L,I,V,A,F,M	**1.000**				-	-	-	-	-	-	-	Y	Q	-	-	-	-	-	-	-	-	S	Y	-	-	-
Patr-B*11:03:01:01	P	L,V,I,A,F,M	0.841				-	-	-	-	-	-	-	Y	Q	-	-	-	G	-	-	-	W	-	Y	-	-	-
Patr-B*11:01	P	L,V,I,A,F,M	0.841				-	-	-	-	-	-	-	Y	Q	-	-	-	G	-	-	-	W	-	Y	-	-	-
**HLA-B*07:02**	**P,A**	**L,V,I,A,F,M**	**1.000**	**B07**	**P**	**L**	-	-	-	-	-	-	-	**Y**	**Q**	-	-	-	-	-	-	-	-	**S**	**Y**	-	-	-
**Papa-B*09:01:01:01**	P	L,V,A,I,F,M	0.839				-	-	-	-	-	-	-	Y	Q	-	-	-	G	-	-	-	W	-	-	-	-	-
Patr-B*28:01	P	A,V,L,I,F,M	0.788				-	-	T	-	-	-	-	F	Q	-	-	-	G	-	-	-	W	-	H	-	-	-
Patr-B*11:02:01:01	P,A	L,V,A,I,F,M	0.813				-	-	-	-	-	-	-	Y	Q	-	-	-	G	-	-	-	W	-	Y	-	-	-
**Papa-B*09:02**	P	L,A,Y,I,F	0.836				-	-	-	-	-	-	-	Y	Q	-	-	-	-	-	-	-	W	-	-	-	-	-
Patr-B*11:04:01:01	P,A	A,L,V,I,M,F,Y	0.783				-	-	-	-	-	-	-	Y	Q	-	-	-	-	-	-	-	W	-	S	-	-	-
Patr-B*19:01	P,A,V,R,T,M	F,Y,I,L,W,M	0.736				-	-	-	-	-	-	-	Y	Q	-	-	-	N	I	A	-	-	-	D	-	-	-
Patr-B*19:02	P,A,V,R,T,M	F,Y,I,L,W,M	0.736				-	-	-	-	-	-	-	Y	Q	-	-	-	N	I	A	-	-	-	D	-	-	-
Patr-B*19:03:01:01	P,A	I,V,L,F	0.765				-	-	-	-	-	-	-	Y	Q	-	-	Y	N	I	A	-	W	-	Y	-	-	-
Patr-B*19:04:01:01	P,A	L,I,F,V,M,W	0.803				-	-	-	-	-	-	-	Y	Q	-	-	-	N	I	A	-	-	-	Y	-	-	-
**Papa-B*01:01**	P,A,M,H	L,M,V,I,F	0.838		P	L	-	-	-	-	-	-	-	C	Q	-	-	-	-	-	-	-	-	-	-	-	-	-
**Papa-B*01:03:01:01**	P,A,M,H	L,M,V,I,F	0.838				-	-	-	-	-	-	-	C	Q	-	-	-	-	-	-	-	-	-	-	-	-	-
**Papa-B*06**	P,A,L,V,M,Q	L,V,M,I,F	0.826				-	-	-	-	-	-	-	C	Q	-	-	-	-	-	-	-	-	-	-	-	-	-
**Papa-B*23:02**	P,A,H,Q	L,I,V,F,M	0.814				-	-	-	-	-	-	-	C	Q	-	-	-	-	-	-	-	-	K	-	-	-	-
**Papa-B*15:02:01:01**	P,A,L,V,I,M	L,I,V,M,F	0.743				-	F	T	M	-	-	-	C	Q	-	-	-	-	-	-	-	-	K	-	-	-	-
**Papa-B*15:03**	P,L,A,M	L,I,V,M	0.723				-	-	T	-	-	-	-	C	Q	-	-	-	-	-	-	-	-	K	-	-	-	-
Patr-B*35:02:01:01	P,A,V,T,I	L,V,I,M	0.772				-	-	A	M	-	-	-	C	Q	-	-	-	-	-	-	-	I	R	Y	-	-	-
Patr-B*12:03:01:01	P,A,V,L,M	L,F,Y,M	0.752				-	-	-	-	-	-	-	Y	S	-	-	-	-	-	-	-	-	R	D	-	-	-
Patr-B*12:02	P,A,R,Q,H,M	L,F,M,Y,V,W	0.757				-	-	-	-	-	-	-	C	Q	-	-	-	-	-	-	-	-	R	D	-	-	-
Patr-B*35:01	P,A,V	L,V,I,A,M,F	0.793				-	-	A	M	-	-	-	Y	Q	-	-	-	-	-	-	-	I	R	Y	-	-	-
**Papa-B*04:01**	P,A	L,I,V,M,F	0.756				-	-	-	-	-	-	-	Y	Q	-	-	-	-	-	-	-	-	-	-	-	S	L
**Papa-B*15:01:01:01**	P,L,A,Q,M	L,I,M,V,F	0.755		P/A	L	-	F	T	M	-	-	-	-	-	-	-	-	-	-	-	-	-	K	-	-	-	-
**Papa-B*04:02**	P,A,H,Q,M	L,M,I,F	0.732				-	-	-	-	-	-	-	C	Q	-	-	Y	-	-	-	-	-	-	-	-	S	L
**Papa-B*08:01:01:01**	A,P,R,V,I,Q,H,M	L,V,M,I,F	0.766				-	-	-	-	-	-	-	C	Q	-	-	-	-	-	-	-	-	-	-	-	-	-
**Papa-B*23:01**	P,A,Q,H,M	L,I,F,V,M	0.823				-	-	-	-	-	-	-	-	-	-	-	-	-	-	-	-	-	K	-	-	-	-
Patr-B*17:01	A,L,P,M,V,Q	L,I,V,M	0.699	**Patr-B*17:01**	A	L	-	-	-	V	-	E	-	F	-	-	-	-	-	-	-	-	-	-	Y	-	S	F
Patr-B*17:02	A,L,P,M,V,Q	L,I,V,M	0.695				-	-	-	V	-	E	-	F	-	-	-	-	-	-	-	-	-	-	Y	-	S	F
**Papa-B*02:01**	P,A,R,H,Q,M	I,L,F,V,M,W	0.761		P/R	I/V	-	-	-	-	-	-	-	C	Q	-	-	-	N	I	A	-	-	-	-	-	-	-
**Papa-B*02:02:01:01**	P,A,R,H,Q,M	I,L,F,V,M,W	0.761				-	-	-	-	-	-	-	C	Q	-	-	-	N	I	A	-	-	-	-	-	-	-
**Papa-B*05**	P,R,A,M,H,Q	I,L,V,F,M,W	0.751				-	-	-	-	-	-	-	C	Q	-	-	-	N	I	A	-	-	-	-	-	-	-
**Papa-B*21:01**	P,A,H,Q	I,L,F,V,M,W	0.735				-	-	-	-	-	-	-	C	Q	-	-	-	N	I	A	-	V	-	-	-	-	-
Patr-B*28:02	R,P,A	V,L,I,A,M	0.801				-	-	T	-	-	-	-	C	Q	-	-	-	G	-	-	-	W	-	H	-	-	-
Patr-B*20:01:02:01	S,T,A	I,V,L	0.901		S/T/A	I/F	-	-	T	M	-	E	N	V	S	-	-	-	N	I	A	-	W	-	-	-	-	-
Patr-B*20:01:01	S,T,A	I,V,L	0.901				-	-	T	M	-	E	N	V	S	-	-	-	N	I	A	-	W	-	-	-	-	-
Patr-B*20:02	S,T,A,V	I,L,V,F	0.881				-	-	T	M	-	E	N	V	S	-	-	-	N	I	A	-	W	-	Y	-	-	-
Patr-B*07:04:01:01	T,S,A,V	I,L,F,V	0.729				-	L	T	M	-	E	N	V	F	F	-	Y	N	I	A	-	W	-	Y	-	-	-
Patr-B*33:01:01:02	A,S,T,G,M	I,L,F,W	0.773				-	L	T	M	-	E	N	V	S	-	-	Y	N	I	A	-	W	-	Y	-	-	-
Patr-B*33:01:01:01	A,S,T,G,M	I,L,F,W	0.773				-	L	T	M	-	E	N	V	S	-	-	Y	N	I	A	-	W	-	Y	-	-	-
Patr-B*07:03:01:01	S,A,T,V,G,M	I,W,F,L	0.719				-	L	T	M	-	E	N	V	S	F	-	Y	N	I	A	-	I	-	Y	-	-	-
Patr-B*07:02:01:01	S,T,A,V,M	I,F,L,W	0.684				-	L	T	M	-	E	N	V	F	F	-	Y	N	I	A	-	I	-	Y	-	-	-
Patr-B*07:06	S,A,T,G,V,M,Y	I,L,F,W	0.748				-	L	T	M	-	E	N	V	S	F	-	Y	N	I	A	-	-	-	Y	-	-	-
Patr-B*07:01	S,A,T,V,G,M	I,L,F,W	0.735				-	L	T	M	-	E	N	V	S	-	-	Y	N	I	A	-	-	-	-	-	-	-
Patr-B*07:05:01:01	S,T,A,V,M,Y	F,L,I,W	0.713				-	L	T	M	-	E	N	V	F	F	-	Y	N	I	A	-	-	-	Y	-	-	-
Patr-B*25:01	S,T,A	I,L,F,W,M	0.799				-	L	T	M	-	E	N	M	S	-	-	Y	N	I	A	-	-	-	-	-	-	-
Patr-B*18:01:01:01	T,S,A,V,M	I,L,F,W,M	0.796		T/S	I/L	-	F	T	M	-	E	N	V	S	-	-	-	N	I	A	-	I	R	S	-	-	-
Patr-B*26:01	T,S,A,V	L,I,V,F,M	0.712				-	-	T	M	-	E	N	V	S	-	-	-	N	T	-	-	-	-	-	-	-	-
Patr-B*10:01	S,T,A	I,L,V	**1.000**				-	-	T	M	-	E	N	M	S	-	-	-	N	I	A	-	W	-	Y	-	-	-
Patr-B*01:01:01:01	S,T,A	I,L,V	**1.000**		S/T	I/L	-	-	T	M	-	E	N	M	S	-	-	-	N	I	A	-	W	-	Y	-	-	-
Patr-B*09:01:01:01	S,T,A	I,L,F,W	0.843				-	-	T	M	-	E	N	M	S	-	-	-	N	I	A	-	I	R	Y	-	-	-
**HLA-B*58:01**	**S,A,T,V**	**W,F,I,Y,M**	**1.000**	**B58**	**S**	**W**	-	-	**A**	**T**	**G**	**E**	**N**	**M**	**S**	-	-	**Y**	**N**	**I**	**A**	-	**I**	**R**	**S**	-	-	-
Patr-B*37:01	T,P,A,V,S	I,L,V,F,M	0.772				-	-	A	M	-	-	G	V	Q	-	-	-	N	I	A	-	I	R	Y	-	-	-
Patr-B*36:01:01:01	T,A,P,V,S	I,L,F,V,M	0.769		T/P/A	I/L/V	-	-	A	M	-	-	G	V	Q	-	-	-	N	I	A	-	I	R	Y	-	-	-
Patr-B*14:01:01:01	P,A,V,T	L,V,I,F,M	0.799				-	-	A	M	-	-	G	I	Q	-	-	-	N	T	-	-	-	S	Y	-	-	-
Patr-B*29:01:01:01	A,V,T,S,I,P,M	V,L,A,I,M	0.761				-	-	A	M	G	-	G	V	Q	-	-	-	-	-	-	-	W	-	Y	-	-	-
Patr-B*06:01	T,A,S,V	L,I,V,M	0.701				-	-	-	M	-	E	N	V	S	-	-	-	-	-	-	-	W	-	Y	-	-	-
Patr-B*06:03:01:01	T,A,S,V	L,I,V,M	0.701				-	-	-	M	-	E	N	V	S	-	-	-	-	-	-	-	W	-	Y	-	-	-
Patr-B*27:01	T,S,A,V	L,I,V,M	0.744				-	-	T	M	-	E	N	V	S	-	-	-	-	-	-	-	-	-	Y	-	-	-

Supplementary Fig. 4 Peptide-binding supertypes of bonobo, chimpanzee, and representative human MHC-B and associated peptide binding pocket composition. Included are all Papa-B (bold font, N=39) and Patr-B (black font, N=85) allotypes deposited in the Immunopolymorphism Database[7] as of July 2022 (and including the six Papa-B newly identified in this study, but not Patr-B*12:01 or *15:01 because their available exon 2 sequences are incomplete or Patr-B*01:02N because it is a null allele), as well as selected human HLA-B allotypes (also in bold black) that are representative of the major peptide-binding supertypes (names given under “Group”; B39 is listed in brackets because there is differing opinion on whether it is a distinct supertype). All allotypes are listed in the top-bottom order corresponding to the MHCcluster[10] order in the heatmap, and horizontal dividing lines correspond to the larger subdivisions of the heatmap and tree clustering (Supplementary Fig. 2). Bonobo and chimpanzee

MHC-B allotypes are summarized by supertype in Supplementary Fig. 3. Shown for each allotype are the amino acid residues that MHCcluster predicts are enriched, in order of most-to-least enriched, at anchor positions 2 (P2) and 9 (P9) of a nonamer peptide. MHCcluster prediction accuracy values < 0.7 are in red and values of 1.0 are in bold. For each allotype grouping, the most preferred P2 and P9 residues are given (when more than one residue is strongly preferred across different allotypes then they are listed and separated by a slash; for some allotypes the second most preferred residue, which is distinguishing between groupings, is given in parentheses). Preferred residues given in blue font are experimentally determined motifs[11], and an asterisk denotes that the observed binding motif differs from the predicted motif[12]. The B and F pockets of an MHC-B molecules bind the P2 and P9 residues, respectively. The MHC-B positions that form each pocket are listed, and positions that are monomorphic within Papa-B and Patr-B are coloured grey. The bonobo Papa-B consensus amino acid for each position is given at the top (bold), with dashes denoting identity to the consensus residue. Allotypes that are assigned to the same supertype group are similarly coloured. Those allotypes that clustered within a major peptide-binding "Group" based on MHCcluster[10] results (Supplementary Fig. 2) but were more appropriately assigned to a different group are coloured accordingly. Two chimpanzee Patr-B with the Patr-B*08:01 binding profile have similarities with HLA-B*15:01 (B62 binding type) in the B pocket and P2 binding profile, as indicated by the shared grey shading. For most of the allotypes that were also assessed by de Groot et al.[12], our assignments agreed. The exceptions are Patr-B*17:01 and 17:02 (darker brown, Patr-B*17:01 supertype) that de Groot et al.[12] assigned to the supertype group designated here at Patr-B*17:03 (lighter brown). We considered those Patr-B part of a distinct group because of their unique B-pocket and distinctive P2 residue preference.

Supplementary Methods

Individual identification

Microsatellite sequences were PCR amplified as described[13] using primers that were modified for MiSeq sequencing by appending adapter sequences (forward adapter: 5′-TCGTCGGCAGCGTCAGATGTGTATAAGAGACAG-3′, reverse adapter: 5′-GTCTCGTGGGCTCGGAGATGTGTATAAGAGACAG-3′) to the 5’ end of each locus-specific forward and reverse primer (Supplementary Table 10). Each of the 13 STR loci and the AMEL gene was amplified in triplicate in a single-step PCR reaction, containing 3–5 µl fecal DNA extract, 2.5 µl 10× AmpliTaq Gold Buffer, 1.75 µl 25 mM MgCl2, 1.5 µl 10 mM dNTPs, 0.5 µl 50 µg/ml BSA, 1.5 µl of 10 mM forward and reverse primers, and 0.25 µl AmpliTaq Gold polymerase (5 U/ml; Applied Biosystems). The thermocycling protocol included an initial denaturation at 94°C for 10 min, followed by 50 cycles of 30 s at 94°C, 30 s at 58°C, and 45 s at 72°C, followed by a final extension at 72°C for 10 min. Triplicate reactions were pooled in equal volume, and 2.5 µl underwent two additional amplifications using the Kapa Biosystems Library Amplification Kit to add MiSeq dual indices and adapters (primers P1: AATGATACGGCGACCACCGA, P2: CAAGCAGAAGACGGCATACGA). Indexed amplicon libraries were pooled for PCR clean-up with Ampure XP Beads (Beckman Coulter) and quantified by the Qubit fluorometer using the dsDNA HS Assay (ThermoFisher). This pooled amplicon library and a PhiX DNA control library (Illumina) were each diluted to a 4 nM DNA concentration, and then the two libraries were pooled in equal volume to achieve a final sequencing library concentration of 20 pM. The library was MiSeq sequenced using 375 bp forward and 51 reverse cycles[13]. The resulting genotypes were determined using the CHIIMP microsatellite allele calling software[13]. Mitochondrial D-loop amplicons were

amplified and sequenced as described for the other loci[13], but in single rather than triplicate reactions and using 2 x 250 bp paired-end reads.

Papa-B *exon 2 and exon 3 genotyping*

The methods for the PCR and Sanger sequencing of exons 2 and 3 of the *Papa-B* gene[4] for samples from TL2-W, TL2-E, and BX, have previously been described[4,14]. Briefly, each exon was separately amplified using primers that anneal to the flanking intronic regions (Supplementary Table 10). The PCR for exon 2 (270 bp) gives a 425 bp product and that for exon 3 (276 bp) gives a 411 bp product. Each exon was amplified at least twice for each sample. The standard exon 2 primers fail to amplify the chimpanzee *Patr-B*17* lineage, due to a 3 bp insertion in the reverse primer site[4,14]. Thus, any sample that genotyped homozygous for exon 2 was subjected to a third *Patr-B*17*-specific PCR. The third PCR uses a *Patr-B*17*-specific reverse primer with the standard exon 2 forward primer and produces a 429 bp amplicon when such an allele is present. As for the other bonobo populations we studied[4], no additional *Papa-B* alleles were amplified by this third PCR, nor were any *Papa-B*17* sequences observed at exon 3. For samples suspected to have a new *Papa-B* allele, exon 2 and exon 3 PCR products were cloned and sequenced from at least one other individual to confirm allele identities (Supplementary Table 1).

Samples from TS, LY, and LI were genotyped using an updated, modified protocol for NGS that follows Illumina's 16S Metagenomic Sequencing Library Preparation protocol. We performed the *MHC-B* exon 2 and exon 3 PCR reactions with the standard locus-specific primers (B2-425 or B3-411) (Supplementary Table 10), but with both forward and reverse primers having the Illumina MiSeq-specific adapter sequences added to their 5' end (forward adapter: 5′-

TCGTCGGCAGCGTCAGATGTGTATAAGAGACAG-3′, reverse adapter: 5′-GTCTCGTGGGCTCGGAGATGTGTATAAGAGACAG-3′). The PCR for each exon from each sample was also performed in triplicate. PCRs were performed in 25 µl reactions with up to 160 ng of total DNA. Reaction mixes for both exons contained, 1 unit of HotStar Taq DNA polymerase and 1x Hot Star Taq Plus buffer (Qiagen), 0.25 mM of each dNTP (Promega), 0.4 µg of bovine serum albumin (BSA; ThermoFisher), and 0.2 µM each of either the MiSeq-adapted NGS-B2-425 or the NGS-B3-411 forward and reverse primers. Added to the exon 2 reaction mix was dimethyl sulfoxide (DMSO; Sigma-Aldrich) to a final concentration of 5%. To the exon 3 reaction mix was added 1 M betaine. PCR cycling for exon 2 consisted of an initial denaturation at 95°C for 5 minutes, followed by touchdown cycling of 95°C for 30 seconds, annealing for 45 seconds, and extension at 72°C for 45 seconds. Annealing began at 69°C and decreased incrementally by 1°C to 66°C, completing two cycles at each temperature. Touchdown was followed by 37 cycles of annealing at 65°C and a final extension of 10 minutes at 72°C. Exon 3 amplifications followed the same touchdown cycling protocol but with annealing starting at 70°C and decreasing by 1°C incrementally to 65°C, followed by 33 cycles of annealing at 57.5°C and a final extension at 72°C for 10 minutes. Reactions were subjected to magnetic bead-based (AMPure XP, Beckman Coulter) PCR cleanup. Triplicates were then pooled by equal volume and the DNA used in an additional PCR to add dual indices (Illumina Nextera XT Index 1 (N7xx) and Index 2 Primers (S5xx) from the Nextera XT Index kit) to amplicons. After a second PCR clean-up, the resulting DNA concentration of each sample was quantified by the Qubit fluorometer using a dsDNA Assay (ThermoFisher). Each sample was then normalized to 4 nM and pooled by equal volume per sample into a single library for sequencing. The final library consisted of equal volumes of exon 2 and exon 3 amplicons (given their similar product size). To increase library complexity and sequence quality, the combined *Papa-B* exon 2 and 3 library was

then pooled in an 80:20 ratio by volume with NGS libraries of different but similar-sized amplicons that were not part of this study, with an additional 20% spike-in of PhiX control library (Illumina). Sequencing was performed using 2 x 300 bp paired-end reads.

Plasmodium screening of faecal samples

The 20 samples collected from TS, LY, and LI were screened for *Plasmodium* sequences as described[1,15]. Briefly, a diagnostic nested PCR was performed that amplifies a 956 bp cytB fragment using first-round primers DW2 (5′-TAATGCCTAGACGTATTCCTGATTATCCAG-3′) and DW4 (5′-TGTTTGCTTGGGAGCTGTAATCATAATGTG-3′) and second-round primers Pfcytb1 (5′-CTCTATTAATTTAGTTAAAGCACA-3′) and PLAS2a (5′-GTGGTAATTGACATCCWATCC-3′). Both reactions were in 25 µl comprised of 0.5 µl dNTPs (10mM of each dNTP), 20 pmol of each primer, 2.5 µl PCR buffer and 0.25 µl Expand Long Template enzyme mix (Roche), with 2.5 µl faecal DNA for the first-round reaction, and 1 µl of the first-round product in the second round. For the first-round PCR cycling, there was an initial denaturation step of 2 min at 94°C, followed by 15 cycles of denaturation (94°C, 10 s), annealing (45°C, 30 s) and elongation (68°C, 1 min), then 35 cycles of denaturation (94°C, 10 s), annealing (48°C, 30 s) and elongation (68°C, 1 min; with 15-s increments for each successive cycle), with a final elongation step of 10 min at 68°C. For the second-round cycling conditions there was an initial denaturation step of 2 min at 94°C, followed by 60 cycles of denaturation (94°C, 10 s), annealing (52°C, 30 s) and elongation (68°C 1 min), followed by a final elongation step of 10 min at 68°C. Each sample was screened using 8-10 faecal DNA aliquots from the same extract to increase the chance of identifying low level infections.

Variation within the **Laverania** *LSA-1 ls6 peptide*

We used limited dilution PCR (also called single genome amplification)[1,2,15] to generate *LSA-1* sequences from existing specimen banks of apes infected with *Laverania* species. DNA extracted from fecal samples was endpoint diluted such that fewer than 30% of PCR reactions for a particular dilution factor yielded an amplified product. A partial *LSA-1* gene region (661bp region containing the C-terminus) was amplified using primers LSA1-F1 (5'-GATGTRTYAGCAGARGATTTATATG-3') and LSA1-R1 (5'-AGTTTCATAAAATATTTAGTTATATCTTCAG-3') in the first round PCR and LSA1-F2 (5'-ACCAGYTATAGAACTTCCATCAGAA-3') and LSA1-R2 (5'-ACGATCTGTAMAATTTCATTGTCTC-3') in the second-round PCR. PCR conditions were as described for *Plasmodium* screening. Endpoint dilution products were MiSeq sequenced as described for microsatellite and mitochondrial amplicons using 2 x 250 paired-end reads.

References

1 Liu, W. *et al.* Wild bonobos host geographically restricted malaria parasites including a putative new *Laverania* species. *Nat. Commun.* **8**, 1-14 (2017).

2 Liu, W. *et al.* African origin of the malaria parasite *Plasmodium vivax*. *Nat. Commun.* **5**, 3346 (2014).

3 Li, Y. *et al.* Eastern chimpanzees, but not bonobos, represent a simian immunodeficiency virus reservoir. *J. Virol.* **18**, 10776-10791 (2012).

4 Wroblewski, E. E. *et al.* Bonobos maintain immune system diversity with three functional types of MHC-B. *J. Immunol.* **198**, 3480-3493 (2017).

5 Sullivan, K. M., Mannucci, A., Kimpton, C. P. & Gill, P. A rapid and quantitative DNA sex test: fluorescence-based PCR analysis of X-Y homologous gene amelogenin. *Biotechniques* **15**, 636-638, 640-631 (1993).

6 Aurrecoechea, C. *et al.* PlasmoDB: a functional genomic database for malaria parasites. *Nucleic Acids Res.* **37**, D539-D543 (2009).

7 de Groot, N. G. *et al.* Nomenclature report 2019: major histocompatibility complex genes and alleles of Great and Small Ape and Old and New World monkey species. *Immunogenet.* **72**, 25-36 (2020).

8 Kumar, S., Stecher, G., Li, M., Knyaz, C. & Tamura, K. MEGA X: molecular evolutionary genetics analysis across computing platforms. *Mol. Biol. Evol.* **35**, 1547 (2018).

9 Hasegawa, M., Kishino, H. & Yano, T.-a. Dating of the human-ape splitting by a molecular clock of mitochondrial DNA. *J. Mol. Evol.* **22**, 160-174 (1985).

10 Thomsen, M., Lundegaard, C., Buus, S., Lund, O. & Nielsen, M. MHCcluster, a method for functional clustering of MHC molecules. *Immunogenet.* **65**, 655-665 (2013).

11 de Groot, N. G. *et al.* AIDS-protective HLA-B* 27/B* 57 and chimpanzee MHC class I molecules target analogous conserved areas of HIV-1/SIVcpz. *PNAS* **107**, 15175-15180 (2010).

12 de Groot, N. G., Stevens, J. M. & Bontrop, R. E. Does the MHC confer protection against malaria in bonobos? *Trends Immunol.* **39**, 768-771 (2018).

13 Barbian, H. J. *et al.* CHIIMP: An automated high-throughput microsatellite genotyping platform reveals greater allelic diversity in wild chimpanzees. *Ecol. Evol.* **8**, 7946-7963 (2018).

14 Wroblewski, E. E. *et al.* Signature patterns of MHC diversity in three Gombe communities of wild chimpanzees reflect fitness in reproduction and immune defense against SIVcpz. *PLoS. Biol.* **13**, e1002144 (2015).

15 Liu, W. *et al.* Origin of the human malaria parasite *Plasmodium falciparum* in gorillas. *Nature* **467**, 420-425 (2010).
