# Supplementary material for: Malaria-driven adaptation of MHC class I in wild bonobo populations
Source: Nat Commun. 2023 Feb 23;14:1033. doi: 10.1038/s41467-023-36623-9 (PMC9950436; doi:10.1038/s41467-023-36623-9)
Supplement: Supplementary file 2 — Reporting Summary [file 41467_2023_36623_MOESM2_ESM.pdf]

nature portfolio

Corresponding author(s): Emily Wroblewski

Last updated by author(s): Feb 16, 2023

Reporting Summary

Nature Portfolio wishes to improve the reproducibility of the work that we publish. This form provides structure for consistency and transparency in reporting. For further information on Nature Portfolio policies, see our Editorial Policies and the Editorial Policy Checklist.

Statistics

For all statistical analyses, confirm that the following items are present in the figure legend, table legend, main text, or Methods section.

n/a	Confirmed	
☐	☒	The exact sample size (*n*) for each experimental group/condition, given as a discrete number and unit of measurement
☐	☒	A statement on whether measurements were taken from distinct samples or whether the same sample was measured repeatedly
☐	☒	The statistical test(s) used AND whether they are one- or two-sided<br>*Only common tests should be described solely by name; describe more complex techniques in the Methods section.*
☒	☐	A description of all covariates tested
☐	☒	A description of any assumptions or corrections, such as tests of normality and adjustment for multiple comparisons
☒	☐	A full description of the statistical parameters including central tendency (e.g. means) or other basic estimates (e.g. regression coefficient) AND variation (e.g. standard deviation) or associated estimates of uncertainty (e.g. confidence intervals)
☐	☒	For null hypothesis testing, the test statistic (e.g. *F*, *t*, *r*) with confidence intervals, effect sizes, degrees of freedom and *P* value noted<br>*Give P values as exact values whenever suitable.*
☒	☐	For Bayesian analysis, information on the choice of priors and Markov chain Monte Carlo settings
☒	☐	For hierarchical and complex designs, identification of the appropriate level for tests and full reporting of outcomes
☒	☐	Estimates of effect sizes (e.g. Cohen's *d*, Pearson's *r*), indicating how they were calculated

Our web collection on statistics for biologists contains articles on many of the points above.

Software and code

Policy information about availability of computer code

Data collection	No software was used.
Data analysis	Statistical analysis was performed using GraphPad QuickCalcs (graphpad.com/quickcalcs/contingency1 (accessed November 2022)) for Fisher's Exact tests, and Medcalc (https://www.medcalc.org/calc/odds_ratio.php (Version 20.215; accessed November 2022)) for odds ratio statistics. Tests of deviation from Hardy-Weinberg equilibrium were performed using Genepop (v. 4.7.5). Plasmodium LSA-1 sequences were assembled using Geneious Prime v. 2022.0.2 (Biomatters, Inc., San Diego, CA, https://www.geneious.com). Mitochondrial haplotype sequences were also analyzed using Geneious Prime 2022.0.2 (https://www.geneious.com), and a phylogenetic tree of mitochondrial sequences was constructed using MEGA X. Microsatellite genotypes were determined using the CHIIMP microsatellite allele calling software (https://github.com/ShawHahnLab/chiimp). Papa-B Sanger sequences were manually inspected using Bioedit (v. 7.2). Papa-B NGS sequence read pairs were assembled using PEAR 0.9.1164 (https://cme.h-its.org/exelixis/web/software/pear/). A de novo assembly of the read pairs was done in Geneious Prime 2022.1.1 (https://www.geneious.com). Peptide-binding supertype specificities and clustering were predicted by MHCcluster (v. 2.0) (https://services.healthtech.dtu.dk/service.php?MHCcluster-2.0).

For manuscripts utilizing custom algorithms or software that are central to the research but not yet described in published literature, software must be made available to editors and reviewers. We strongly encourage code deposition in a community repository (e.g. GitHub). See the Nature Portfolio guidelines for submitting code & software for further information.

Data

Policy information about availability of data

All manuscripts must include a data availability statement. This statement should provide the following information, where applicable:

- Accession codes, unique identifiers, or web links for publicly available datasets
- A description of any restrictions on data availability
- For clinical datasets or third party data, please ensure that the statement adheres to our policy

New Papa-B sequences generated in this study have been deposited in GenBank under accession codes MW039484-MW039487 and ON936818-ON936819 (https://www.ncbi.nlm.nih.gov/genbank/) and were also deposited in the IPD-MHC database (https://www.ebi.ac.uk/ipd/mhc/group/NHP/). New bonobo mitochondrial DNA and LSA-1 sequences generated in this study have also been deposited in GenBank under accession codes ON936815-ON936817 and OM570838-OM570859, respectively. Previously identified LSA-1 sequences used in this study are available in PlasmoDB (https://plasmodb.org), and sequence IDs are provided in the Supplementary Information. Source data are provided with this paper in either the Supplementary Information or Source Data file.

Human research participants

Policy information about studies involving human research participants and Sex and Gender in Research.

Reporting on sex and gender	*Use the terms sex (biological attribute) and gender (shaped by social and cultural circumstances) carefully in order to avoid confusing both terms. Indicate if findings apply to only one sex or gender; describe whether sex and gender were considered in study design whether sex and/or gender was determined based on self-reporting or assigned and methods used. Provide in the source data disaggregated sex and gender data where this information has been collected, and consent has been obtained for sharing of individual-level data; provide overall numbers in this Reporting Summary. Please state if this information has not been collected. Report sex- and gender-based analyses where performed, justify reasons for lack of sex- and gender-based analysis.*
Population characteristics	*Describe the covariate-relevant population characteristics of the human research participants (e.g. age, genotypic information, past and current diagnosis and treatment categories). If you filled out the behavioural & social sciences study design questions and have nothing to add here, write "See above."*
Recruitment	*Describe how participants were recruited. Outline any potential self-selection bias or other biases that may be present and how these are likely to impact results.*
Ethics oversight	*Identify the organization(s) that approved the study protocol.*

Note that full information on the approval of the study protocol must also be provided in the manuscript.

Field-specific reporting

Please select the one below that is the best fit for your research. If you are not sure, read the appropriate sections before making your selection.

☐ Life sciences ☐ Behavioural & social sciences ☒ Ecological, evolutionary & environmental sciences

For a reference copy of the document with all sections, see nature.com/documents/nr-reporting-summary-flat.pdf

Ecological, evolutionary & environmental sciences study design

All studies must disclose on these points even when the disclosure is negative.

Study description	This study is of wild bonobos (Pan paniscus) from 12 locations within their range in the Democratic Republic of the Congo. From these populations 392 faecal samples were analysed from a minimum of 174 bonobos (genetically identified by available Papa-B, microsatellite, and mitochondrial D-loop sequences), 137 and 64 newly analysed for this study, respectively. For the 7 populations that were well-sampled (5 or more bonobos), we compared MHC-B polymorphism across these populations according to whether they were endemically infected (2 populations (TL2-W and TL2-E) or not (5 populations)) with Plasmodium parasites related to those parasites that cause malaria disease in humans. TL2-W and TL2-E are physically divided by the Lomami River which has been shown to prevent gene flow. MHC-B polymorphism was characterized for the two exons (2 and 3) which encode the polymorphic peptide binding domain of the protein and are both highly polymorphic. Of the 12 total sites, 6 were previously characterized, and 6 were newly characterized for this study. This study has a factorial design in that a bonobo population was categorized according to Plasmodium presence or absence and its location relative to the Lomami River (either west or east).
Research sample	We focused on bonobos (Pan paniscus) as a study species because they, along with chimpanzees, are the closest living relatives of humans and because they harbor Plasmodium parasites closely related to the Plasmodium falciparum parasite that afflicts African populations. In addition, the Plasmodium infection pattern among bonobos, in which TL2-W and TL2-E harbor infection while other populations do not, contrasts that of chimpanzees that do not show such a clear spatial divide in infection. The spatial division of infection among bonobo populations enabled us to compare MHC-B polymorphism between infected and uninfected bonobos.

The research sample included 174 wild bonobos (392 faecal samples) from 12 populations from within the bonobo range, which is exclusively in the Demoratic Republic of the Congo (Africa). This sample of 12 populations is meant to represent the range of genetic diversity present among wild bonobos (as a species), particularly considering populations on both sides of the Lomami River, which is a known geographic barrier to bonobo gene flow. 59 bonobos were from TL2 bonobos (where Plasmodium is present) and 115 from other sites (where Plasmodium is not present); 64 bonobos (137 faecal samples) from 5 sites (TL2: 59, BX: 1, TS: 2 LY: 17, LI: 1)), were newly analyzed for this study, while 110 bonobos (255 faecal samples) were previously characterized and included here. Because these are wild populations that are not habituated to (i.e., used to) human presence, and the fecal samples used as the source of DNA for this study were collected opportunistically from the forest floor, we do not have any information, such as age or sex, about the bonobo fecal sample donors. Sex was determined genetically for the 20 samples collected most recently at TS, LY, and LI, and all sample donors (4 bonobos) were identified as male.

Sampling strategy	Because 5 uninfected bonobo populations located west of the Lomami River were characterized previously, we targeted characterizing samples available from 1) from the infected population (TL2) on both sides of the Lomami River (TL2-W and TL2-E) and 2) other populations located East of the river. The faecal samples collected from these populations were collected previously for other studies, so we obtained aliquots from as many genetically distinguishable bonobos as possible and at least 2 samples per bonobo whenever possible. Because these are limited-size and natural, not captive, populations, we did not pre-determine sample size or do a sample size calculation – we included as many individuals as possible.
Data collection	Of the primary data collectors/generators: E.E.W., A.G.A., S.E.H., and L.A.G. generated and analyzed the Papa-B sequence data, with sequences analyzed in either BioEdit or Geneious and sample genotypes recorded in Excel; E.E.W. performed the MHCcluster analysis with supertypes recorded in Excel; W.L. conducted the LSA-1 sequencing with sequences analysed and stored in Geneious. Y.L. and A.J.C. generated and analyzed the mitochondrial and microsatellite genotype data for TS, LY, and LI, using Geneious and CHIIMP, respectively.
Timing and spatial scale	Faecal samples were collected at the 12 sites over the following years, based on the ability to conduct field expeditions: TL2 (West and East), Oct. 2012-Feb. 2013; BX, Oct. 2016; TS/LYI: Jun.-July 2017; LI: Nov. 2017; BJ, Jun. 2006; KR, Sept. 2006- Jan. 2007; BN, July-Aug. 2010; IK, May 2010 (June 2010)-11; LK, Mar.-May 2006; ML, Jan.-May 2010. Populations for sampling and inclusion in this study were chosen according to their location within the range (to get a representative sample across their range) and for their difference in Plasmodium endemicity (TL2 being the only bonobo site known to have endemic Plasmodium). The bonobo range exists entirely within the Democratic Republic of the Congo, but with bonobos living both west of and east of the Lomami River. Five sites (BJ, BN, LK, IK, KR, ML) were characterized previously, and therefore available to include in this study, but all except one (BJ) were located west of the Lomami River, and none of them harbor malaria infection. Therefore, we targeted additional populations for this study to include the Plasmodium infected bonobos at TL2 (TL2-W and TL2-E) and additional sites east of the Lomami River, where only two bonobos had been characterized previously. The new data for this study were generated between 2018 and 2022.
Data exclusions	Data from 5 sites (BJ, BX, TS, LY, and LI) were included/reported among the total population genetic data, but they were not included in the population comparisons based on Plasmodium infection because so few samples, and therefore so few bonobos, were represented (fewer than our defined threshold of 5 bonobos, which represents a minimum of 10% of a bonobo community).<br><br>For seven bonobos (6 at the TL2-E site, and 1 at site TS, both located east of the Lomami River), the second Patr-B allele in the genotype was not able to be assigned and were conservatively designated “Unknown”. Only single samples were available from five of these bonobos, and ambiguous sequences were observed in at least two reactions per exon. The other two bonobos (TL2-07 and TL2-40.1 in TL2-East) are heterozygous for exon 2 of Papa*B-01:01/3 and an exon 2 sequence shared by Papa-B*01:02, 04:01, 09:02, all three of which were otherwise present among TL2-East bonobos. Both these bonobos are homozygous for Papa-B*01:01/3 in exon 3. We could not resolve if this was due to a failure in amplification of a second exon 3 or the presence of a novel recombinant allele. Resolving this ambiguity will require sequencing the allele in different heterozygous combinations. Currently, the allele is assigned as “Unknown”.
Reproducibility	For each of the two exons of Papa-B, for each sample, PCR and sequencing was performed at least twice (i.e., each sample had at least 4 PCR amplifications). This repetition, along with confirmation of sequences by cloning ensures sequence and genotype accuracy. In addition, at least two samples per bonobo were included, whenever more than 1 sample was available. The single bonobo sampled at BX was PCR-genotyped and found to be homozygous by both Sanger as well as NGS methods.
Randomization	Randomization was not part of this study because it is a study of natural, not experimental, populations. We could only include the populations to which we had access, and identify populations as having Plasmodium infection or not and being west or east of the Lomami River.
Blinding	Allele sequencing and identification was done blind to the Plasmodium infection status of the sample/animal, and the status was only assigned after genotyping was completed.

Did the study involve field work? ☒ Yes ☐ No

Field work, collection and transport

Field conditions	All samples for this study were collected previously as part of another study, and this study simply used aliquots of those archived samples for a source of DNA. However, for the previous sample collection faecal samples left by bonobos (i.e., collected non-invasively) were collected into RNAlater nucleic acid preservative (1:1 vol/vol), and they were first stored and transported at ambient temperature and subsequently stored at -80°C after reaching the U.S.A.
Location	Democratic Republic of the Congo (initial sample collection), U.S.A. (long-term sample storage)

Access & import/export	At the time, all samples were obtained with permission from the D.R.C. Ministries of Scientific Research and Technology, the Ministries of Health and Environment, and the National Ethics Committee, and the Department of Ecology and Management of Plant and Animal Resources at the University of Kisangani as previously reported10,39. Shipment of samples was done in compliance with the regulations of the Convention on International Trade in Endangered Species of Wild Fauna and Flora and with governmental export and import permits from the D.R.C. and U.S.A.
Disturbance	Since the faecal samples were collected non-invasively, after being desposited by the animal, there was minimal to no disturbance to the animals. Because this study utilized aliquots of samples collected non-invasively, the study was not classified as animal research by the Stanford Administrative Panel on Laboratory Animal Care or the Institutional Animal Care and Use Committee at Washington University in St. Louis.

Reporting for specific materials, systems and methods

We require information from authors about some types of materials, experimental systems and methods used in many studies. Here, indicate whether each material, system or method listed is relevant to your study. If you are not sure if a list item applies to your research, read the appropriate section before selecting a response.

Materials & experimental systems

n/a	Involved in the study
☒	☐ Antibodies
☒	☐ Eukaryotic cell lines
☒	☐ Palaeontology and archaeology
☐	☒ Animals and other organisms
☒	☐ Clinical data
☒	☐ Dual use research of concern

Methods

n/a	Involved in the study
☒	☐ ChIP-seq
☒	☐ Flow cytometry
☒	☐ MRI-based neuroimaging

Animals and other research organisms

Policy information about studies involving animals; ARRIVE guidelines recommended for reporting animal research, and Sex and Gender in Research

Laboratory animals	The study did not involve laboratory animals.
Wild animals	Wild animals were studied non-invasively by collecting faecal samples after they were deposited by the animal.
Reporting on sex	For most of the study samples, sex of the bonobo sample donor was unknown. Because these are wild populations that are not habituated to (i.e., used to) human presence, and the fecal samples used as the source of DNA for this study were collected opportunistically from the forest floor. Therefore, sample collectors were not able to observe the bonobo sample donors to identify their sex. Sex was determined genetically for the 20 samples collected most recently at TS, LY, and LI, and all sample donors (4 bonobos) were identified as male. Sex was not considered as part of the study design because sampling of bonobos at the study sites was only able to be done opportunistically, and analyses were performed at the population-level. Papa-B genotypes of bonobos within populations are not expected to be biased by sex.
Field-collected samples	Faeces were collected into RNAlater (1:1 vol/vol) for nucleic acid preservation. Samples were first stored and transported at ambient temperature and subsequently stored at -80°C after reaching the U.S.A.
Ethics oversight	Because this study utilized aliquots of samples collected non-invasively, the study was not classified as animal research by the Stanford Administrative Panel on Laboratory Animal Care or the Institutional Animal Care and Use Committee at Washington University in St. Louis.

Note that full information on the approval of the study protocol must also be provided in the manuscript.
